# Landscape quality drives ecological responses to habitat loss and fragmentation

**DOI:** 10.1038/s41559-026-03095-1

**Published:** 2026-06-17

**Authors:** Robert J. Fletcher, Thomas A. H. Smith, Maggie Jones, Fredericke Schellenberg, Rikki Payne, Nicholas Kortessis, Emilio M. Bruna, Robert D. Holt

**Affiliations:** 1 Department of Zoology, Conservation Research Institute, University of Cambridge, Cambridge, UK; 2 Department of Wildlife Ecology and Conservation, University of Florida, Gainesville, FL USA; 3 Department of Integrative Biology, University of Wisconsin-Madison, Madison, WI USA; 4 Department of Biology, Wake Forest University, Winston Salem, NC USA; 5 Center for Latin American Studies, University of Florida, Gainesville, FL USA; 6 Department of Biology, University of Florida, Gainesville, FL USA

**Keywords:** Conservation biology, Population dynamics

## Abstract

Studies have suggested that the quality of the lands surrounding habitat patches can modify the effects of habitat loss and fragmentation on species and influence biodiversity predictions across regions. As landscape matrices tend to be complex and vary with habitat change, isolating such effects is challenging. Here we disentangle the effects of habitat loss, fragmentation and surrounding landscape quality in a large, multiscale manipulative experiment on a plant–herbivore system. We find that habitat loss, fragmentation and surrounding matrix quality all affect survival rates, with the greatest negative effects of fragmentation and lower matrix quality under high habitat loss. Demographic rate changes resulted in strong negative effects of habitat loss, fragmentation and low matrix quality on population size at the landscape scale. Our findings indicate that the benefits of high landscape quality are greater in landscapes with low habitat fragmentation, contesting the common expectation that the surrounding matrix matters only in the most fragmented landscapes. This underscores that the quality of the surrounding landscape can have outsized effects on biodiversity in remaining habitats.

## Abstract (web summary)

Habitat loss and fragmentation are among the greatest threats to biodiversity. Here the authors show that habitat quality of the surrounding landscape can also affect demographic rates and population size, especially where habitat loss and fragmentation are low.

## Main

Habitat loss is a globally pervasive threat to biodiversity, leading to the extinction of species and disruptions to the functioning of ecosystems^1,2^. It is often accompanied with ‘habitat fragmentation’ where the remaining habitat becomes broken apart, leading to a greater number of small and more isolated patches^3,4^. Whether habitat fragmentation has similar detrimental effects on biodiversity remains a contentious issue because it is often challenging to isolate fragmentation effects from that of habitat loss^5–10^. It has long been argued that habitat fragmentation has negative effects on biodiversity as a result of fragmentation leading to declines in patch size and increases in isolation^1,11^. Nonetheless, these negative effects often attributed to fragmentation could actually be due to habitat loss, because these studies rarely compared different ways in which the same total habitat loss is ‘broken apart’^7^.

Progress towards reconciling these disparate perspectives hinges on overcoming three primary obstacles. The first is our limited understanding of how the landscape-scale effects of variation in patch (or fragment) size, number and isolation—including potentially synergistic ones—are driven by the surrounding landscape ‘matrix’ in which fragments are embedded^12–14^. Variation in the surrounding landscape matrix can alter landscape quality, leading to shifts in the composition of biological communities, altered rates of mortality and changes in the likelihood of movements between habitats^15^. The quality of the landscape matrix is fundamental to interpreting the value of land sharing versus land sparing, and it can alter predictions for biodiversity loss across landscapes and regions^16,17^. Recent syntheses also suggest that the effects of the type of surrounding landscape matrix on species occurrence or extinction risk could potentially exceed those of patch size and isolation^13^ or even habitat loss^14^.

The second hurdle is deciding how best to disentangle the relative effects of habitat loss, fragmentation and landscape matrix characteristics on biodiversity^12,18^. Most studies to date have gathered observational data in naturally fragmented landscapes^10,13,19^. Although this has the advantage of documenting ecological responses at realistic spatial scales, these landscapes rarely include all combinations of the factors of interest and the age and history of fragments and matrix are often unknown^8^. Habitat loss and fragmentation can also catalyse a decline in matrix quality in the surrounding landscape^20,21^, making it challenging to isolate the effects of each. For instance, ongoing deforestation in the tropics often coincides with increases in habitat fragmentation and greater land-use intensification, where the matrix surrounding remaining forest fragments becomes less hospitable^22–24^. These shortcomings can be overcome with an experimental approach, which also allows for precise tests of predictions from theory^25^. However, fragmentation experiments beyond the mesocosm scale are rarely tractable, and the few landscape-scale experiments that have been conducted rarely manipulate both fragment properties (for example, size and isolation) and the type or quality of matrix in which fragments are embedded^1,15^.

**Fig. 1:**
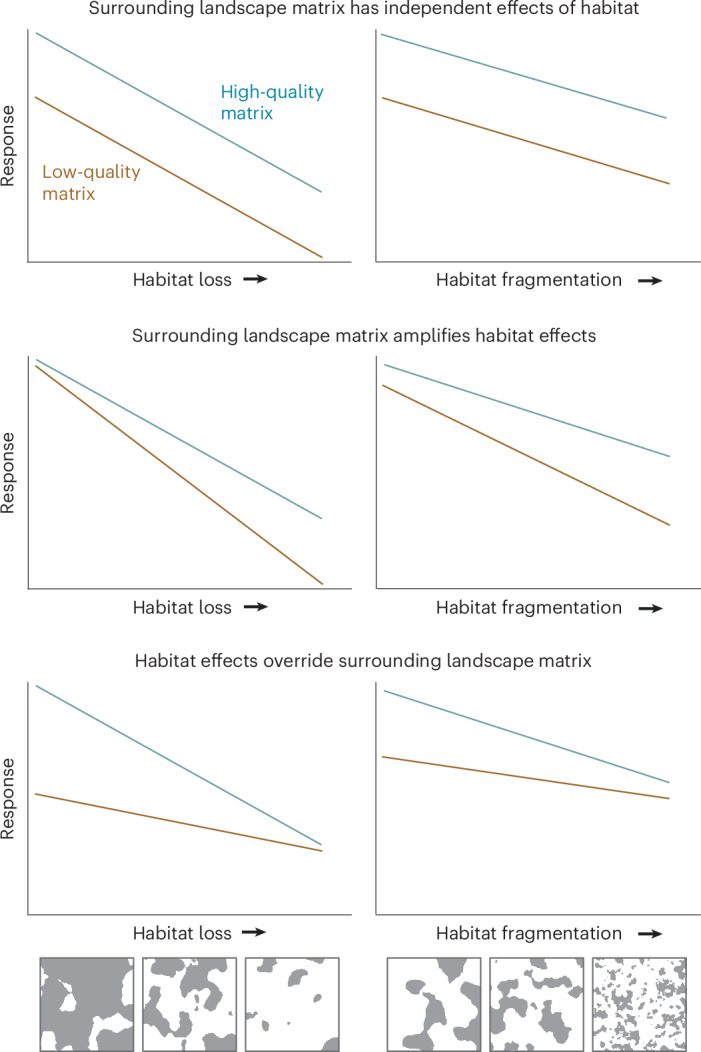
Alternative predictions for how the surrounding landscape matrix may alter effects of habitat fragmentation. The surrounding landscape matrix has been predicted to have three different types of effects on habitat loss and fragmentation: effects may be additive and independent of loss and fragmentation, low matrix quality can amplify effects of loss and fragmentation, or high loss and fragmentation may override any effects of the matrix. Shown are predictions when increasing loss or fragmentation has negative effects on organisms. In general, predictions for loss and fragmentation are often similar, although the magnitude of expected effects may be greater for habitat loss.

Finally, conceptual models often make contradictory or even incompatible predictions for how the surrounding landscape matrix influences biodiversity. For instance, reductions in matrix quality are often hypothesized to exacerbate the effects of both habitat loss and fragmentation^14,18,24,26^. As habitat loss and fragmentation increase, organisms are increasingly exposed to the matrix environment, such that a decline in quality can lead to greater impacts of loss and fragmentation on survival^27^ (Fig. 1). Yet low matrix quality may have greater impacts with increasing loss than fragmentation as an increase in loss leads to greater amounts of matrix in landscapes^28^. Alternatively, the effects of high habitat loss or fragmentation could overwhelm any matrix effects by limiting carrying capacities of populations or by selection reducing the likelihood that individuals disperse through the matrix^18,29^ (Fig. 1).

**Fig. 2:**
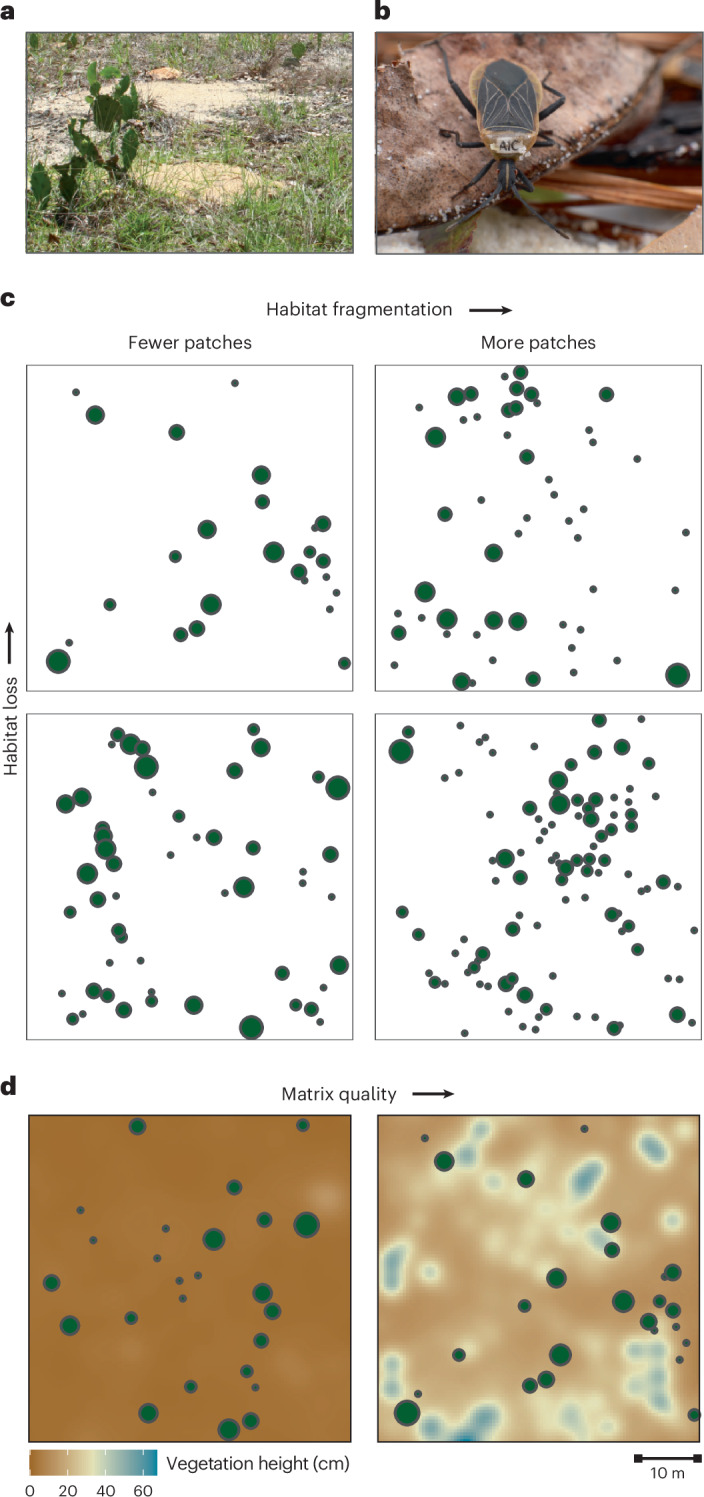
A multiscale experimental design to test the effects of habitat loss, fragmentation and matrix quality. **a**, *Opuntia* cactus is patchy habitat for many arthropods and it is surrounded by a landscape matrix that can vary in quality for cactus-specializing insects. **b**, A marked, adult *C. vittiger*, a cactus specialist and crop pest, moving through the landscape matrix. **c**, Examples of landscapes where habitat loss and fragmentation (number of patches for a given amount of loss) were manipulated by altering cactus habitat. Points are cactus patches and point size is proportional to patch size. All landscapes were initiated with the same pretreatment habitat amount, patch-size distribution and abundance of *C. vittiger*. **d**, The quality of surrounding matrix for a *C. vittiger* was manipulated in half the landscapes that received habitat manipulations (*n* = 12) by reducing vegetation height. Photographs by R.J.F. (**a**) and Christine W. Miller (**b**).

To test these alternative predictions, we manipulated habitat loss, fragmentation and surrounding matrix quality in 27 experimental landscapes comprising over 3,000 patches of habitat used by wild populations of a specialist insect herbivore, *Chelinidea vittiger*, which is a pest on *Opuntia* cactus (Fig. 2, Supplementary Table 1 and Supplementary Fig. 1). To our knowledge, this is the largest experiment that has manipulated both habitat and matrix characteristics, with landscapes being over 12 times larger and sampling running over 17 times longer than other prior experiments (Supplementary Table 2). We also conducted a review and meta-analysis of other landscape experiments that have manipulated both the matrix and some component of habitat to determine the generality of habitat loss, fragmentation and matrix effects on species living in fragmented landscapes.

## Results

### Multiscale experiment

We designed a landscape experiment to factorially manipulate habitat loss, fragmentation and matrix quality, as well as changes in patch-size distributions, over time. In our design, high-loss treatments resulted in half the remaining habitat relative to low-loss treatments. Similarly, for a given amount of habitat loss, our low-fragmentation treatments had approximately half the number of patches remaining in landscapes in comparison to high-fragmentation treatments (Fig. 2c and Supplementary Table 1). For matrix treatments, we contrasted unmanipulated surrounding landscapes (high-quality matrix) to those where we reduced vegetation height surrounding habitat patches, which was previously shown to increase mortality rates^30^, thus creating a low-quality landscape matrix. We then tracked populations using mark–recapture techniques over time. We fit superpopulation models^31^ to mark–recapture data from 3,357 individuals marked over 4 years post-treatment (approximately 12 generations), which enabled estimating population size and demography (survival and recruitment) of *C. vittiger* across treatments at the landscape scale, while accounting for imperfect detection.

### Interactions of habitat loss, fragmentation and the landscape matrix

**Fig. 3:**
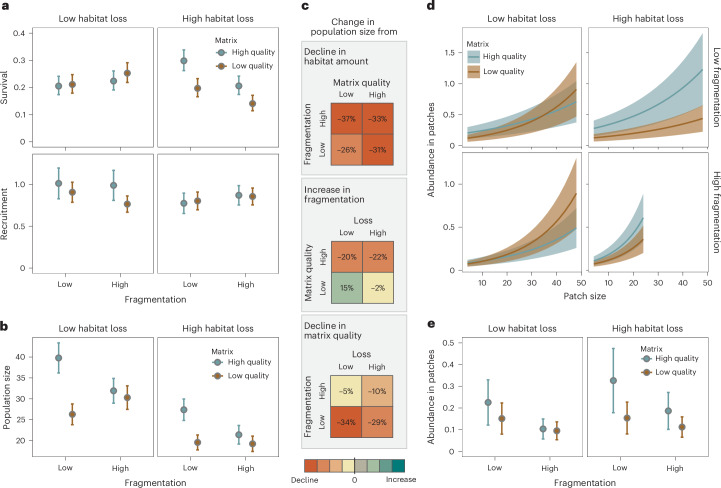
Effects of habitat loss, fragmentation and matrix quality at two spatial scales on demography and population size of *C. vittiger* (mean ± s.e.m.) across experimental landscapes. **a**,**b**, Landscape-scale manipulations (*n* = 3 per factorial treatment combination) on mean estimated demographic rates of monthly survival and per capita recruitment (**a**) and population size (±s.e.m.) from mark–recapture data (**b**). **c**, The estimated percentage population decline from increasing loss, increasing fragmentation and a decline in matrix quality. **d**,**e**, Patch-scale changes in predicted mean abundance (±s.e.m.) as a function of patch size (**d**) and average patch size by landscape treatment (**e**). Number of patches per plot varied on the basis of landscape treatments (low loss, low fragmentation—*n* = 51; low loss, high fragmentation—*n* = 100; high loss, low fragmentation—*n* = 25; high loss, high fragmentation—*n* = 51).

Among several models with varying possible effects of our experimental treatments (Supplementary Table 3), the most supported model included interactive effects of habitat loss, fragmentation and matrix treatments, along with monthly and annual trends, on both survival and the probability of entry, a parameter used to estimate recruitment (Supplementary Table 4). On the basis of the most supported model, there were strong main effects of habitat loss and matrix quality on survival, as well as interactions of habitat loss with both fragmentation and matrix quality treatments (Supplementary Table 5). Survival rates declined with greater fragmentation and lower matrix quality, but only at high, and not low, habitat loss (Fig. 3a). The magnitude of interactions for fragmentation and the matrix on survival was similar (Supplementary Table 5). There were positive effects of matrix quality on the probability of entry, as well as a negative interaction of habitat fragmentation with matrix quality treatments (Supplementary Table 5); however, the combination of these effects led to weak effects of treatments on per capita recruitment (Fig. 3a). At the landscape scale, changes in demographic rates resulted in strong negative effects of habitat loss, fragmentation and eroded matrix quality on population size (Fig. 3b). High habitat loss led to 26–37% declines in population size, whereas an increase in fragmentation, doubling the number of fragments, led to 20–22% declines in population size, but only when fragments were surrounded by high-quality matrix (Fig. 3c). A reduction in matrix quality led to 29–34% declines in population size, similar in magnitude to habitat loss impacts and greater than fragmentation, but only in less fragmented landscapes (Fig. 3c).

We then estimated effects at the scale of habitat patches on abundance of *C. vittiger* as a function of patch size and assessed how treatments may alter the patch-size effect using a zero-inflated, Bayesian *N*-mixture model^32^, using detectability estimates from our superpopulation model as priors. At the patch scale, high fragmentation reduced local abundance, whereas abundance increased with patch size (Fig. 3d and Supplementary Table 5). There were interactions of patch size with habitat loss and fragmentation, as well as three-way interactions with matrix quality, habitat loss and fragmentation. In general, effects of landscape treatments on population abundance at the patch scale were largely similar to effects on population size at the landscape scale (Fig. 3b,e).

To contextualize these experimental effects, we scanned the literature for experimental studies that manipulated both matrix characteristics and habitat. Of 2,221 articles we reviewed, only 7 articles (5 unique experiments) manipulated both the matrix and some component of habitat (Supplementary Table 2). Therefore, these results (Supplementary Note 1 and Supplementary Figs. 4–8) should be treated with caution. Briefly, the results overall appear to support our experimental findings of interactive effects of fragmentation and surrounding matrix quality (Supplementary Fig. 4), but the effect sizes were highly variable (Supplementary Table 3), probably reflecting the heterogeneity in matrix and fragmentation treatments relative to the organisms and ecological processes of focus.

## Discussion

When experimentally isolating the role of habitat loss, fragmentation and surrounding matrix quality, we find that each of these factors can have detrimental effects on populations. These results run counter to claims that habitat fragmentation may be inconsequential, or even beneficial, for biodiversity relative to habitat loss^3,33^. Our experiment illustrated that the surrounding landscape matrix can have large effects, but that these effects interact with fragmentation, where positive effects of matrix quality were greater in landscapes with low habitat fragmentation. These results challenge the expectation that the matrix matters only in the most fragmented landscapes^14,18,24^.

Despite long-standing interest in habitat loss, fragmentation and the landscape matrix, we found surprisingly few experiments that have manipulated both habitat and the matrix to interpret how the surrounding landscape may alter effects of habitat loss and fragmentation (Supplementary Table 2). Effect sizes across these experiments nonetheless highlight that observed effects of the landscape matrix tended to be as strong or stronger than that of loss or fragmentation and these effects interacted with fragmentation. As noted, these effect sizes were nonetheless highly variable. The limited number of experiments that have manipulated both habitat and the matrix makes it challenging to generalize habitat–matrix relationships, particularly because the experiments tended to focus primarily on abundance responses, arthropods and grassland ecosystems. More experiments that manipulate both habitat and the surrounding landscape for different taxonomic groups and ecosystems, and focus on community-level responses, are needed.

Our experiment adds to these prior works in three ways. First, by estimating population vital rates hypothesized to drive effects of the matrix on populations, we show that the surrounding matrix impacts populations by altering survival^15^. Theory consistently emphasizes that matrix effects may be driven by mortality arising during dispersal, which leads to a ‘dispersal filter’ across landscapes^15,18,34^. Previous research with *C. vittiger* using short-term experiments identified that matrix conditions with low vegetation height result in greater mortality^30^ and that such effects are greater based on matrix conditions immediately adjacent to remaining habitat^35^. Our experiment adds to our previous work^30,35–37^ in this system by directly testing for the role of matrix quality relative to habitat loss and fragmentation to interpret their joint and potentially interacting effects, and by diagnosing the demographic rates that drive population change. Second, by testing for both patch- and landscape-scale responses, our results highlight that effects are largely consistent across scales, in that negative effects of fragmentation and matrix quality on abundances in patches and population size across entire landscapes both occurred (Fig. 2). One key difference was the role of habitat loss: at the landscape scale, high loss led to lower population sizes, but effects on abundances at the patch scale were weak. This disparity probably reflects the fundamental difference that at the patch-scale, investigations, including ours, typically focus on distribution and abundance in the remaining habitat, while at the landscape-scale focus is often on wholesale loss of habitat on total population size^18^. Third, our study design was driven by the scale of dispersal and population dynamics in this species^36,37^, such that results are probably meaningful to other specialist species inhabiting fragmented landscapes^38,39^. Experiments in large landscapes (for example, extents relevant for dispersal) and for long durations are needed to capture how landscape characteristics impact ecological processes^36^ and the lasting impacts of land change on biodiversity^1^. Although our results provide key insights for wild populations, it remains to be seen if similar patterns arise at the community level, in terms of both species interactions and biodiversity metrics.

Spatial ecology theory and previous fragmentation experiments have played large roles in interpreting landscape change and its applications to conservation^1,25^, yet theory on how the matrix alters outcomes has remained less developed and poorly tested^15^. Some models have predicted that a decline in matrix quality could increase effects of habitat loss^18^ and fragmentation^40,41^ (Fig. 1). In contrast to this theory, in our experiment we found that with high fragmentation, there was little effect of matrix quality on population size. In highly fragmented landscapes, mean patch size was smaller (Supplementary Table 1) and these small patches may be below a threshold for local population suitability, such that even though small patches may act as stepping stones for movement^42^, ultimately fewer individuals are expected to disperse and be impacted by the matrix than in less fragmented landscapes. In a similar way, selection against dispersal can arise in highly fragmented landscapes^29,43^, which would reduce the impact of matrix quality on dispersers. Recent debate on the value of small patches has not yet grappled with the problem of what size is too small for biodiversity^44,45^. Our results call for more quantitative evaluations to identify at what size patches become too small to support ecological function. We contend that limiting habitat loss while fostering connectivity among remaining habitats by improving the landscape matrix are complementary and effective strategies for mitigating ongoing environmental change^46^.

## Methods

### Multiscale experiment

We conducted this experiment at the Ordway-Swisher Biological Station (OSBS; 29.4° N, 82.0° W) in central Florida, USA. The cactus bug *C. vittiger* (Hemiptera: Coreidae) is dependent upon prickly pear cactus (*Opuntia* spp.), where it feeds, breeds and aggregates throughout its life. In central Florida, *C. vittiger* uses *Opuntia mesacantha* (formerly *Opuntia humifusa*) primarily in relatively open, grass-dominated, old field and sandhill habitats that contain limited canopy cover. By focusing on a habitat specialist entirely dependent on *Opuntia*, we manipulated habitat in a field setting without common concerns regarding appropriate habitat delineation. The vegetation surrounding patches of Opuntia in OSBS consist of graminoids, such as wiregrass (*Aristida stricta*) and broom sedge (*Andropogon* spp.), forbs, predominantly dogfennel (*Eupatorium capillifolium*) and narrow-leaf silk grass (*Pityopsis graminifolia*), and scattered woody species consisting of gopher apple (*Geobalanus oblongifolius*), pawpaw (*Asimina incana*) and brambles (*Rhubus* spp.).

In the study area, female *C. vittiger* generally begin producing and depositing eggs on or near *Opuntia* in early March and continue egg laying through September, with two to three generations per year. There are five nymph stages before eclosion to adults^47^. Nymphs cannot fly and typically reside at the oviposition site until reaching the adult stage. Adults are winged, but rarely fly; instead, adult cactus bugs typically walk between cactus patches through a matrix that is unsuitable for reproduction^48^. Movements of adults are thus relatively localized and can be easily tracked via mark–recapture techniques^36,38^.

Between 2018 and 2022, we conducted a field experiment that manipulated habitat loss, fragmentation and matrix quality. To do so, we first identified 27 landscapes of 50 × 50 m^2^ with appropriate habitat arranged in six geographic blocks. All landscapes were >50 m apart within blocks. We aimed for landscape size to be relevant to dispersal processes in this species; previous results showed that average dispersal distances range between 5 m and 13 m, such that landscape size was ~5–10× greater than expected dispersal distances. We then cleared all plots of existing cactus. We planted cactus in each plot between June 2018 and October 2018. For each landscape, we planted 120 patches of cactus of similar quality and the same amount (number of patches and cactus segments), for a total of 1,800 patches across landscapes. Cactus patches were >1 m apart.

Spatial distribution of patches was based on realizations of a Thomas cluster point process, fit to mapped data of natural distributions in the study area^49^. This resulted in aggregated distributions of cactus that mimicked naturally patchy cactus distribution in the study area. We varied the patch-size distribution of cactus on the basis of the number of cactus pads, where size distribution varied on the basis of previously observed natural size distributions, ranging from 4 to 52 cactus pads per patch. We bred *C. vittiger* (November 2017 to June 2018) in the greenhouse and released them in the field in July 2018 (50 *C. vittiger* per landscape).

In May to June 2019, we then manipulated cactus via habitat loss and fragmentation treatments on 24 of the 27 landscapes, leaving 3 landscapes as controls. We had two treatments for habitat loss applied at the landscape scale: low and high, where for low loss 35% of habitat area (cactus pads) was removed and for high loss 70% of habitat area was removed (Supplementary Table 1). We had two treatments for habitat fragmentation at the landscape scale: for low fragmentation, we preferentially removed small patches, leaving a greater portion of large patches remaining. For high fragmentation, we preferentially removed large patches of cactus. Relative to the 120 patches in controls, these treatments resulted in the following combinations: low loss–low fragmentation (51 patches remained), low loss–high fragmentation (100 patches remained), high loss–low fragmentation (25 patches remained) and high loss–high fragmentation (51 patches remained; Supplementary Table 1). Finally, we randomly selected half the treatment landscapes (*n* = 12) to manipulate matrix quality by mowing three times per year (May, August and October). A prior tethering experiment showed that survival rate of *C. vittiger* was lower in matrices with low vegetation height^30^, such that we interpret mowed landscapes as representing low-quality matrix. From June 2019 to June 2022, we surveyed all remaining patches within each landscape every 3 weeks except over winter (and during COVID lockdown), when bugs are dormant. Across the entire study, we conducted 3 pretreatment surveys and 28 post-treatment surveys.

We modelled individual encounter histories of marked *C. vittiger* using a superpopulation model (also known as the POPAN model), an extension of the Jolly–Seber model for capture–mark–recapture data. This modelling framework enabled estimates of apparent survival, recruitment and population size while accounting for imperfect detection. The full model tested for habitat loss, fragmentation and matrix quality as fixed effects on apparent survival (*φ*), the probability of entry (*γ*, which is used to derive recruitment) and superpopulation size of adult *C. vittiger* while accounting for temporal variation in imperfect detection (*p*). We also included month to account for seasonal variation and year as a continuous covariate to account for potential trends. We tested the best fit among several models featuring different combinations of effects (described in Supplementary Table 3, along with their interpretation) using Akaike information criteria corrected for sample size (AICc). We found the strongest explanatory model using ΔAICc assuming ΔAICc > 2 was sufficient to distinguish the most supported model(s).

To estimate effects at the patch scale, we modelled population abundance in patches using a Bayesian state–space formulation, where we assessed the effects of habitat loss, fragmentation, matrix quality and patch size on abundance in patches. We focused on a factorial model of treatment effects and their interactions (considering up to three-way interactions), as we expected that each of these landscape treatments could interact with patch size to drive abundance outcomes. We treated the probability of detecting a marked adult, *p*, as a nuisance variable, allowing it to vary among survey periods *t*. We modelled the post-treatment abundance, *N*_*tjk*_, of adult *C. vittiger* at the patch scale during survey time *t* at patch *k* in landscape *j*, by considering population size as a latent variable in a zero-inflated, binomial *N*-mixture model as follows:

$${N}_{{tjk}} \sim {\mathrm{Poisson}}({\omega \lambda }_{{tjk}}),$$

$${y}_{{tjk}} \sim {\mathrm{binomial}}({N}_{{tjk}},{p}_{t}),$$

$$\log \left({\lambda }_{{tjk}}\right)=X{\boldsymbol{\beta }}+{\delta }_{j},$$

where $\omega$ is a parameter that describes the zero inflation, ${\lambda }_{{tjk}}$ is the expected value for abundance, ${y}_{{tjk}}$ is the observed abundance, *X* is a covariate matrix that includes patch size (log-transformed), habitat loss, fragmentation and matrix quality (including two- and three-way interactions), ${\boldsymbol{\beta }}$ is a vector of parameters being estimated for covariates and$\,{\delta }_{j}$ is a random effect of landscape *j*. We assumed vague priors for all fixed parameters on a log scale (mean = 0, s.d. = 10) and used uniform hyperpriors for standard deviation parameters (mean = 0, s.d. = 10). We used estimates of *p*_*t*_ from the POPAN model as fixed priors, assuming a beta distribution. We ran all models in jags using the jagsUI package to call jags from R. We ran four chains for 37,500 Markov chain Monte Carlo iterations and thinned chains by 50 after a burn-in of 15,000 and an adaptation phase of 15,000, ultimately saving 5,000 samples from the posteriors. We assessed model convergence using the Gelman–Rubin statistic R-hat, assuming that an R-hat > 1.05 indicated convergence problems.

### Review and meta-analysis

We reviewed literature on habitat loss, fragmentation and the matrix to identify experiments that manipulated both habitat and the matrix and measured effects on biodiversity at the individual, population or community level. Our goal was to understand the extent to which these key elements of the landscape have been experimentally tested, given theoretical expectations for the matrix impacting the outcome of habitat loss and fragmentation effects^18,27,41^. While observational studies on the matrix have been synthesized and suggest that the matrix can strongly modify patch-size and habitat fragmentation effects^14^, such effects are challenging to interpret because matrix conditions can often be confounded with habitat amount and quality across landscapes^20^.

We used Web of Science to search for experiments that manipulated habitat and the matrix to determine the number of experiments, what aspects of the matrix and habitat were manipulated and identify the extent to which the matrix alters outcomes of habitat loss and fragmentation. On 22 September 2024, we searched for articles using the search phrase: experiment* AND (matrix OR “edge contrast” OR “edge type*” OR “land use” OR “land-use”) AND (habitat* OR fragment*) AND (ecolog* OR conserv* OR landscape* OR seascape*). The first part of this phrase isolates experiments, the second and third parts use terms that are relevant to the matrix and habitat and the fourth uses terms that aim to identify ecological studies (as opposed to experiments in other fields). On the basis of this search, we identified 2,221 investigations. We then screened these articles to determine if the matrix and habitat were manipulated in some way (for example, land-cover change, altering resources such as perches and so on), resulting in the selection of seven articles that met these criteria (Supplementary Table 2). These articles manipulated habitat amount, fragmentation based on changing either the number of patches or patch isolation, and matrix type (simple binary changes, typically of ‘low’ and ‘high’ quality; Supplementary Table 2).

For each article, we extracted the mean and variance for all measured response variables for each experimental treatment (all combinations of matrix and habitat manipulations). Response variables included measures of abundance, biomass, diversity and movement. Two articles included responses based on ecological function (decomposition and herbivory), but we did not consider them here. We extracted these data from the results sections of the articles and from figures using the metaDigitise package^50^. We also recorded the sample size for each treatment group.

We calculated effect sizes using Hedges’ *g*, which is a measure of standardized difference in means that corrects for small sample sizes^51^. Effect sizes and variances were calculated for main effects and interactions in factorial studies, or ‘focal’ effect sizes, as well as pairwise interactions^52,53^. To estimate the precision of effect sizes, we calculated the variance of each effect. We calculated matrix effect sizes as the difference between the sum of the mean responses in all experimental landscapes with one type of matrix and the sum of mean responses in landscapes with the alternative matrix type. We calculated this difference such that a positive effect size indicated that the higher quality matrix, as defined by the authors of each article (in studies that did not define matrix quality, we considered the matrix type most dissimilar to habitat as lower quality^15^), had a positive effect on the response variable. Habitat amount effect sizes were calculated similarly as the difference in the summed mean responses in landscapes with less habitat and landscapes with relatively more habitat. In this case, a positive effect size indicated that greater habitat amount in a landscape had a positive effect. For habitat fragmentation effects, we calculated the difference between responses in fragmented compared with unfragmented landscapes, such that a positive effect indicated an increased response in unfragmented landscapes. Studies manipulated habitat fragmentation in two ways: (1) by altering the number of patches for a given habitat amount (subdivision); or (2) by altering the effective isolation of patches, either based on changes in distance between patches or by connecting patches with corridors (thereby reducing effective isolation). We estimated effect sizes for measures of abundance and diversity such that a positive effect indicated that the given measure was greater in landscapes with higher quality matrix, more habitat or less fragmentation. For movement, only one type of metric was used in the selected studies, immigration rates (mean number of immigrants per patch), and we estimated these effect sizes as positive when immigration was higher in the ‘better’ landscape.

To understand patterns in responses to manipulations of the matrix and habitat area and fragmentation, we ran multilevel meta-analysis models using the rma.mv function in the metafor package in R^54^. We assessed the influence of matrix and habitat manipulations and their interactions on effect sizes using a meta-regression model with Hedges’ *g* as the response variable, which was weighted by precision, landscape treatment (matrix, habitat amount, habitat fragmentation or any pairwise interaction) as a moderating factor and a random slope of landscape element by study to account for heterogeneity within landscape elements^55^. We tested the overall significance of landscape treatments using the *Q*_M_ statistic, which describes the amount of heterogeneity in effect sizes explained by the model^51^. We assessed model diagnostics using funnel plots (Supplementary Fig. 6) and by identifying potential outliers using Cook’s distance, hat values and studentized residuals^56^ (Supplementary Fig. 7). To identify extreme values based on these metrics, we used a chi-square threshold of 50% of the distribution to identify outliers for Cook’s distance, 2 × (7/*N*) threshold for hat values (where *N* is the number of effect sizes and three for studentized residuals^56^. We then re-ran models removing these effect sizes to determine the sensitivity of estimates to their inclusion (Supplementary Fig. 8).

### Reporting summary

Further information on research design is available in the Nature Portfolio Reporting Summary linked to this article.

## Supplementary information

Supplementary Information Supplementary Note 1, Tables 1–7 and Figs. 1–8.

Reporting Summary

Peer Review File

## Data Availability

The data used in this study are available via figshare at 10.6084/m9.figshare.30508496 (ref. ^57^).

## Code availability

The code used to prepare the data and complete the analyses in this study are available via figshare at 10.6084/m9.figshare.30508496 (ref. ^57^).

## References

[1] Haddad, N. M. et al. Habitat fragmentation and its lasting impact on Earth. *Sci. Adv.* **1**, e1500052 (2015). 26601154 10.1126/sciadv.1500052 PMC4643828

[2] Wilcove, D. S., Rothstein, D., Dubow, J., Phillips, A. & Losos, E. Quantifying threats to imperiled species in the United States. *Bioscience* **48**, 607–615 (1998).

[3] Fahrig, L. Effects of habitat fragmentation on biodiversity. *Annu. Rev. Ecol. Evol. Syst.* **34**, 487–515 (2003).

[4] Fletcher, R. J. Jr et al. Addressing the problem of scale that emerges with habitat fragmentation. *Glob. Ecol. Biogeogr.* **32**, 828–841 (2023).

[5] Diamond, J. M. The island dilemma: lessons of modern biogeographic studies for the design of natural reserves. *Biol. Conserv.* **7**, 129–146 (1975).

[6] Simberloff, D. & Abele, L. G. Refuge design and island bioegeographic theory: effects of fragmentation. *Am. Nat.* **120**, 41–50 (1982).

[7] Fahrig, L. Ecological responses to habitat fragmentation per se. *Annu. Rev. Ecol. Evol. Syst.* **48**, 1–23 (2017).

[8] Fletcher, R. J. Jr et al. Is habitat fragmentation good for biodiversity? *Biol. Conserv.* **226**, 9–15 (2018).

[9] Chase, J. M., Blowes, S. A., Knight, T. M., Gerstner, K. & May, F. Ecosystem decay exacerbates biodiversity loss with habitat loss. *Nature* **584**, 238–243 (2020). 32728213 10.1038/s41586-020-2531-2

[10] Gonçalves-Souza, T. et al. Species turnover does not rescue biodiversity in fragmented landscapes. *Nature* **640**, 702–706 (2025). 40074894 10.1038/s41586-025-08688-7

[11] Pfeifer, M. et al. Creation of forest edges has a global impact on forest vertebrates. *Nature* **551**, 187–191 (2017). 29088701 10.1038/nature24457 PMC5681864

[12] Bender, D. J. & Fahrig, L. Matrix structure obscures the relationship between interpatch movement and patch size and isolation. *Ecology* **86**, 1023–1033 (2005).

[13] Prugh, L. R., Hodges, K. E., Sinclair, A. R. E. & Brashares, J. S. Effect of habitat area and isolation on fragmented animal populations. *Proc. Natl Acad. Sci. USA* **105**, 20770–20775 (2008). 19073931 10.1073/pnas.0806080105 PMC2634894

[14] Ramirez-Delgado, J. P. et al. Matrix condition mediates the effects of habitat fragmentation on species extinction risk. *Nat. Commun*. **13**, 595 (2022). 10.1038/s41467-022-28270-3 PMC8807630 35105881

[15] Fletcher, R. J. Jr et al. The prominent role of the matrix in ecology, evolution, and conservation. *Annu. Rev. Ecol. Evol. Syst.* **55**, 423–447 (2024).

[16] Koh, L. P. & Ghazoul, J. A matrix-calibrated species–area model for predicting biodiversity losses due to land-use change. *Conserv. Biol.* **24**, 994–1001 (2010). 20214672 10.1111/j.1523-1739.2010.01464.x

[17] Phalan, B., Onial, M., Balmford, A. & Green, R. E. Reconciling food production and biodiversity conservation: land sharing and land sparing compared. *Science* **333**, 1289–1291 (2011). 21885781 10.1126/science.1208742

[18] Yamaura, Y., Fletcher, R. J., Lade, S. J., Higa, M. & Lindenmayer, D. From nature reserve to mosaic management: improving matrix survival, not permeability, benefits regional populations under habitat loss and fragmentation. *J. Appl. Ecol.* **59**, 1472–1483 (2022).

[19] Zhang, H. L., Chase, J. M. & Liao, J. B. Habitat amount modulates biodiversity responses to fragmentation. *Nat. Ecol. Evol*. **8**, 1437–1447 (2024). 10.1038/s41559-024-02445-1 38914711

[20] Haynes, K. J. & Cronin, J. T. Confounding of patch quality and matrix effects in herbivore movement studies. *Landsc. Ecol.* **19**, 119–124 (2004).

[21] Ries, L., Fletcher, R. J., Battin, J. & Sisk, T. D. Ecological responses to habitat edges: mechanisms, models, and variability explained. *Annu. Rev. Ecol. Evol. Syst.* **35**, 491–522 (2004).

[22] Hansen, M. C. et al. The fate of tropical forest fragments. *Sci. Adv*. **6**, eaax8574 (2020). 10.1126/sciadv.aax8574 PMC7065873 32195340

[23] Hansen, M. C. et al. Global land use extent and dispersion within natural land cover using Landsat data. *Environ. Res. Lett.* **17**, 034050 (2022).

[24] Leite, M. D., Boesing, A. L., Metzger, J. P. & Prado, P. I. Matrix quality determines the strength of habitat loss filtering on bird communities at the landscape scale. *J. Appl. Ecol.* **59**, 2790–2802 (2022).

[25] Resasco, J., Bruna, E. M., Haddad, N. M., Banks-Leite, C. & Margules, C. R. The contribution of theory and experiments to conservation in fragmented landscapes. *Ecography* **40**, 109–118 (2017).

[26] Galán-Acedo, C. & Fahrig, L. The effects of fragmentation per se on patch occupancy are stronger and more positive in a landscape with a higher quality and more homogeneous matrix. *Ecography* **2025**, e07462 (2025).

[27] Fahrig, L. How much habitat is enough?. *Biol. Conserv.* **100**, 65–74 (2001).

[28] Fahrig, L. Effect of habitat fragmentation on the extinction threshold: a synthesis. *Ecol. Appl.* **12**, 346–353 (2002).

[29] Schtickzelle, N., Mennechez, G. & Baguette, M. Dispersal depression with habitat fragmentation in the bog fritillary butterfly. *Ecology* **87**, 1057–1065 (2006). 16676549 10.1890/0012-9658(2006)87[1057:ddwhfi]2.0.co;2

[30] Fletcher, R. J. Jr. et al. Towards a unified framework for connectivity that disentangles movement and mortality in space and time. *Ecol. Lett.* **22**, 1680–1689 (2019). 31347244 10.1111/ele.13333

[31] Schwarz, C. J. & Arnason, A. N. A general methodology for the analysis of capture–recapture experiments in open populations. *Biometrics* **52**, 860–873 (1996).

[32] Kery, M. & Royle, J. A. *Applied Hierarchical Modeling in Ecology:**Analysis of Distribution, Abundance and Species Richness in R and BUGS* p. 783 (Academic, 2016).

[33] Fahrig, L. Relative effects of habitat loss and fragmentation on population extinction. *J. Wildl. Manag.* **61**, 603–610 (1997).

[34] Vandermeer, J. & Carvajal, R. Metapopulation dynamics and the quality of the matrix. *Am. Nat.* **158**, 211–220 (2001). 18707319 10.1086/321318

[35] Smith, T. A. H., Holt, R. D., Bruna, E. M. & Fletcher, R. J., Jr. Isolating the role of the matrix at patch and landscape scales. *J. Anim. Ecol*. **94**, 1800–1810 (2025). 10.1111/1365-2656.70089 PMC12424283 40556444

[36] Fletcher, R. J. Jr., Reichert, B. & Holmes, K. The negative effects of habitat fragmentation operate at the scale of dispersal. *Ecology* **99**, 2176–2186 (2018). 30112822 10.1002/ecy.2467

[37] Fletcher, R. J., Smith, T. A. H., Kortessis, N., Bruna, E. M. & Holt, R. D. Landscape experiments unlock relationships among habitat loss, fragmentation, and patch-size effects. *Ecology* **104**, e4037 (2023). 36942593 10.1002/ecy.4037

[38] Fletcher, R. J. Jr., Acevedo, M. A., Reichert, B. E., Pias, K. E. & Kitchens, W. M. Social network models predict movement and connectivity in ecological landscapes. *Proc. Natl Acad. Sci. USA* **108**, 19282–19287 (2011). 22084081 10.1073/pnas.1107549108 PMC3228428

[39] Fletcher, R. J. Jr. et al. Network modularity reveals critical scales for connectivity in ecology and evolution. *Nat. Commun.* **4**, 2572 (2013). 24096937 10.1038/ncomms3572

[40] Fahrig, L. When does fragmentation of breeding habitat affect population survival?. *Ecol. Model.* **105**, 273–292 (1998).

[41] Chetcuti, J., Kunin, W. E. & Bullock, J. M. Matrix composition mediates effects of habitat fragmentation: a modelling study. *Landsc. Ecol.* **36**, 1631–1646 (2021).

[42] Fletcher, R. J. Jr., Acevedo, M. A. & Robertson, E. P. The matrix alters the role of path redundancy on patch colonization rates. *Ecology* **95**, 1444–1450 (2014). 25039208 10.1890/13-1815.1

[43] Cheptou, P. O., Hargreaves, A. L., Bonte, D. & Jacquemyn, H. Adaptation to fragmentation: evolutionary dynamics driven by human influences. *Phil. Trans. R. Soc. B*. **372**, 20160037 (2017). 10.1098/rstb.2016.0037 PMC5182433 27920382

[44] Riva, F. & Fahrig, L. The disproportionately high value of small patches for biodiversity conservation. *Conserv. Lett.* **15**, e12881 (2022).

[45] Fahrig, L. Why do several small patches hold more species than few large patches?. *Glob. Ecol. Biogeogr.* **29**, 615–628 (2020).

[46] Brodie, J. F. et al. A well-connected Earth: the science and conservation of organismal movement. *Science* **388**, eadn2225 (2025). 40273266 10.1126/science.adn2225

[47] DeVol, J. E. & Goeden, R. D. Biology of *Chelinidea vittiger* with notes on its host–plant relationships and value in biological weed control. *Environ. Entomol.* **2**, 231–240 (1973).

[48] Schooley, R. L. & Wiens, J. A. Movements of cactus bugs: patch transfers, matrix resistance, and edge permeability. *Landsc. Ecol.* **19**, 801–810 (2004).

[49] Fletcher, R. J. Jr. & Fortin, M. J. *Spatial Ecology and Conservation Modeling: Applications With R* (Springer, 2018).

[50] Pick, J. L., Nakagawa, S. & Noble, D. W. A. Reproducible, flexible and high-throughput data extraction from primary literature: the metaDigitise R package. *Methods Ecol. Evol.* **10**, 426–431 (2019).

[51] Koricheva, J., Gurevitch, J. & Mengersen, K. *Handbook of Meta-Analysis in Ecology and Evolution* (Princeton Univ. Press, 2013).

[52] Macartney, E. L., Lagisz, M. & Nakagawa, S. The relative benefits of environmental enrichment on learning and memory are greater when stressed: a meta-analysis of interactions in rodents. *Neurosci. Biobehav. Rev.* **135**, 104554 (2022). 10.1016/j.neubiorev.2022.104554 35149103

[53] Gurevitch, J., Morrison, J. A. & Hedges, L. V. The interaction between competition and predation: a meta-analysis of field experiments. *Am. Nat.* **155**, 435–453 (2000). 10753073 10.1086/303337

[54] Viechtbauer, W. Conducting meta-analyses in R with the metafor package. *J. Stat. Softw.* **36**, 1–48 (2010).

[55] Rubio-Aparicio, M. et al. Testing categorical moderators in mixed-effects meta-analysis in the presence of heteroscedasticity. *J. Exp. Educ.* **88**, 288–310 (2020).

[56] Viechtbauer, W. & Cheung, M. W. L. Outlier and influence diagnostics for meta-analysis. *Res. Synth. Methods* **1**, 112–125 (2010). 26061377 10.1002/jrsm.11

[57] Fletcher, R. J., Jr. Data from Landscape quality drives ecological responses to habitat loss and fragmentation. *figshare*10.6084/m9.figshare.30508496 (2026). 10.1038/s41559-026-03095-1 PMC13346112 42310138

