# Supplementary material for: Landscape quality drives ecological responses to habitat loss and fragmentation
Source: Nat Ecol Evol. 2026 Jun 17;10(7):1265–72. doi: 10.1038/s41559-026-03095-1 (PMC13346112; doi:10.1038/s41559-026-03095-1)
Supplement: Supplementary file 1 — Supplementary Note 1, Tables 1–7 and Figs. 1–8. [file 41559_2026_3095_MOESM1_ESM.pdf]

nature ecology & evolution

Supplementary information

https://doi.org/10.1038/s41559-026-03095-1

Landscape quality drives ecological responses to habitat loss and fragmentation

In the format provided by the authors and unedited

Table of Contents

Supplementary Note 1: Review and meta-analysis 2

Table S1. Experimental design highlighting the patch-size distribution and treatments implemented. 4

Table S2. Summary of experiments that have manipulated habitat and matrix structure. 5

Table S3. Alternative model structures considered for survival and the probability of entry 6

Table S4. Contrasting models for estimating superpopulation size, apparent survival (ϕ), and the probability of entry (γ) 7

Table S5. Parmeter estimates (SE, 95% lower and upper confidence intervals) from the two most supported* superpopulation, mark-recapture models 8

Table S6. Parmeter estimates (SD, 95% lower and upper credible intervals) from a hierarchical N-mixture model testing for the effects of landscape treatments, patch size, and their interactions on patch abundance. 9

Table S7. Estimates of heterogeneity in the meta-analysis focused on treatment effects. 10

Fig. S1. Examples of habitat and matrix treatments across landscapes over time. 11

Fig. S2. Estimated survival and probability of entry (+/- SEM) over time 12

Fig. S3. Estimated population size (+/- SEM) of adults over time 13

Fig. S4. Estimates from meta-analysis of experiments testing the effects of the matrix relative to habitat loss and fragmentation based on ‘focal’ and ‘pairwise’ effect sizes 14

Fig. S5. Raw focal effect sizes (Hedge’s g) taken from seven studies and 140 responses 15

Fig. S6. Contour-enhanced funnel plot of the standard error and residuals 16

Fig. S7. Diagnostics for focal effect sizes 17

Fig. S8. Sensitivity analysis for focal effect sizes..18

Supporting References 19

Supplementary Note 1: Review and meta-analysis

To contextualize the results from our multi-scale experiment, we scanned the literature for experimental studies that manipulated both matrix characteristics and habitat. Of 2221 articles we reviewed, only seven articles (five unique experiments) manipulated both the matrix and some component of habitat (Table S2). These experiments manipulated habitat (amount and/or fragmentation) and the matrix (matrix type, typically inferred as high versus low quality) at landscape scales, yet no experiments manipulated habitat loss over time, measured demographic or community rates that theory emphasizes as mechanisms for effects [1,2], or tested for cross-scale interactions with patch size. Based on these experiments, studies more frequently found evidence for matrix effects (main effects or interactions; *N* = 6; 86%) than habitat amount (*N* = 3, 42%) or fragmentation (*N* = 4; 57%). Similar patterns occurred for individual tests within studies (amount: *N* = 47, 17%; fragmentation: *N* = 56, 18%; matrix: *N* = 66, 27%).

Despite a limited number of experiments, we obtained 140 'focal' effect sizes [3] (Supplementary Fig. S4) from these manipulations, with abundance (*N* = 128), diversity (*N* = 8), and movement (*N* = 4) being measured. Using meta-regression, we first contrasted a model where loss, fragmentation and matrix treatments were moderators of effect sizes and contrasted that model to one where 'fragmentation' was split into studies manipulating the number of patches (subdivision) versus isolation (distance between patches or corridors)[4]. Overall, there was support for distinguishing subdivision and isolation rather than pooling into simply 'fragmentation' ($\chi^2 = 9.77$, $P = 0.008$). In general, main effects of habitat amount and number of patches were weak, whereas isolation and matrix quality had large estimates but had high heterogeneity

(Supplementary Table S7; Fig. S5), leading an overall lack of significance. However, we found evidence that the effects of habitat fragmentation interacted with both habitat amount and matrix quality (Fig. S4). To interpret these interactions, we calculated pair-wise effect sizes [3] (N = 240). These pair-wise effect sizes highlighted the effects of fragmentation (number of patches) reversed with habitat amount: effects were negative with low habitat amount but tended positive with high habitat amount (Fig. S4b). Pair-wise effect sizes suggested that positive effects of matrix quality were slightly higher when fragmentation was low (number of patches) than when it was high, similar to the results of the multi-scale experiment (Fig. 3b, e), although this interaction appeared weaker based on pair-wise effect sizes than with focal effect sizes (Fig. S4b).

Funnel plots did not suggest strong evidence of publication bias (Fig. S6). These results were mostly not sensitive to potential extreme values (Supplementary Fig. S7), although removing outliers identified based on studentized residuals led to significant main effects of isolation and matrix treatments (Supplementary Fig. S8).

Table S1. Experimental design highlighting the patch-size distribution and treatments implemented. For each combination, there were 3 replicates. Pre-treatment landscapes started with 1156 cactus pads (i.e., segments or cladodes) distributed across 120 patches. With low loss, 752 pads remained whereas for high loss, 344 pads remained, each of which were distributed across different patch size distributions reflecting low and high fragmentation (number of patches for a given habitat amount). Each treatment combination was replicated 6 times, with 3 replicates having a matrix treatment and the other 3 replicates being a matrix control.

Patch size	Pre-treatment	Low loss (~35% loss of habitat)		High loss (~70% loss of habitat)	
(# pads)	(control)	Low frag	High frag	Low frag	High frag
4	61	15	58	8	31
8	24	8	22	4	11
12	12	7	10	4	5
16	10	8	6	3	3
24	6	6	2	3	1
32	4	4	1	2	0
48	3	3	1	1	0
**Landscape summary metrics (SE):**					
Total number of patches	120	51	100	25	51
Average patch size	9.63	14.75	7.52	13.76	6.75
Nearest neighbor distance (m)	2.41 (0.03)	3.36 (0.07)	2.26 (0.06)	5.13 (0.30)	3.88 (0.12)

Notes: Total number of patches and patch size were manipulated as part of treatments (so no SEs are reported). Nearest neighbor distances measure the distance to the next closest patch.

Table S2. Summary of experiments that have manipulated habitat and matrix structure. From a review of 2221 articles, we found 5 experiments (7 articles) that manipulated the matrix and habitat at the landscape scale to test for potential habitat loss and fragmentation effects interacting with the surrounding matrix.

			Treatment manipulations							Responses		
	Expt #	Aquatic/ terrestrial	Loss	Frag	Patch size	Matrix	*N*	Time frame	Landscape size	Taxa	Type	Scale*
Astrom and Part [5]	1	terrestrial	NA	isolation (corridors)	N	Type**	96	103 days	0.00004 ha	Microarthropods	Guild abundance, richness	Patch
Diekotter et al. [6]	2	terrestrial	amount	#patches	Y	Type	36	< 1 month	0.02 ha	Arthropods	pollinator Activity, abundance of certain species	Landscape
Haynes and Crist [7]	2	terrestrial	amount	#patches	Y	Type	36	6 weeks	0.02 ha	Arthropods	Herbivory	Landscape
Haynes et al. [8]	2	terrestrial	amount	#patches	Y	Type	36	4 weeks	0.02 ha	Arthropods	species density	Landscape
Goodsell and Connell [9]	3	aquatic	NA	isolation (distance)	NA	Type	48	3 days	0.0001 ha	Arthropods	Guild presence, composition	Patch
Goodwin and Fahrig [10]	4	terrestrial	amount	#patches	NA	Type	64	6 weeks	0.0025 ha	Arthropods	Patch immigration	Patch
Spiesman et al. [11]***	5	terrestrial	amount	isolation (distance)	Y	Type	24	1 month	0.0001 ha	Bacteria	Species richness, composition	Patch
This experiment	6	terrestrial	Loss	#patches	Y	Quality	27	5 years	0.25 ha	Arthropods	Demography, abundance	Patch, Landscape

**Patch scale responses reflect summaries of individual patches within landscapes. Landscape-scale responses reflect summaries pooled across patches within landscapes*

***Assumed to reflect quality*

****Effect of habitat amount and patch size tests were equivalent based on study design (authors reported 'patch size')*

Table S3. Alternative model structures considered for survival and the probability of entry, which is used to estimate recruitment. All models allowed detection probability to vary by month and year and the superpopulation size to vary as a function of Loss × Frag × Matrix. For all treatment interactions, we only considered pairwise interactions (not higher order interactions).

**Survival (ϕ)**	**Description / Rationale**
ϕ(.)	Survival is constant over time and across treatments
ϕ(Trend + month)	Survival varies by month and increases or decreases across years
ϕ(Loss + Frag)	Habitat loss and fragmentation treatments influence survival
ϕ(Loss + Frag + Matrix)	Habitat loss, fragmentation and matrix treatments influence survival
ϕ(Loss × Frag)	Habitat loss and fragmentation treatments interact to influence survival
ϕ(Loss × Frag × Matrix)	Habitat loss, fragmentation and matrix treatments interact to influence survival
ϕ(Loss + Frag + Trend + month)	Habitat loss and fragmentation treatments influence survival and survival varies by month and increases or decreases across years
ϕ(Loss + Frag + Matrix+ Trend + month)	Habitat loss, fragmentation and matrix treatments influence survival, and survival varies by month and increases or decreases across years
ϕ(Loss × Frag + Trend + month)	Habitat loss, fragmentation and matrix treatments interact to influence survival, and survival varies by month and increases or decreases across years
ϕ(Loss × Frag × Matrix + Trend + month)	Habitat loss, fragmentation and matrix treatments interact to influence survival, and survival varies by month and increases or decreases across years
**Probability of entry (γ)**	
γ(Trend + month)	Recruitment varies by month and increases or decreases across years
γ(Loss + Frag + Trend + month)	Habitat loss and fragmentation treatments influence recruitment, and recruitment varies by month and increases or decreases across years
γ(Loss × Frag + Trend + month)	Habitat loss, fragmentation and matrix treatments interact to influence recruitment, and recruitment varies by month and increases or decreases across years
γ(Loss + Frag + Matrix + Trend + month)	Habitat loss, fragmentation and matrix treatments influence recruitment, and recruitment varies by month and increases or decreases across years
γ(Loss × Frag × Matrix + Trend + month)	Habitat loss, fragmentation and matrix treatments interact to influence recruitment, and recruitment varies by month and increases or decreases across years

Table S4. Contrasting models for estimating superpopulation size, apparent survival (ϕ), and the probability of entry (γ), which is related to recruitment. Only models within 10 AICc units are shown.

Model	K	AICc	ΔAICc	AICc weight	Deviance
ϕ(Loss × fragmentation × matrix + trend + month)<br>γ(Loss × fragmentation × matrix + trend + month)	51	5244.92	0.00	0.48	-13007.9
ϕ(Loss × fragmentation × matrix + trend + month)<br>γ (Loss + fragmentation + matrix + trend + month)	48	5245.37	0.45	0.39	-13001.3
ϕ(Loss × fragmentation + trend + month)<br>γ (Loss × fragmentation × matrix + trend + month)	48	5249.1	4.18	0.06	-12997.5
ϕ(Loss × fragmentation × trend + month)<br>γ (Loss + fragmentation + matrix + trend + month)	45	5250	5.08	0.04	-12990.5
ϕ(Loss × fragmentation × trend + month)<br>γ (Loss × fragmentation × matrix + trend + month)	50	5252.03	7.11	0.01	-12998.7
ϕ(Loss × fragmentation × trend + month)<br>γ (Loss + fragmentation + matrix + trend + month)	47	5252.91	7.99	0.01	-12991.7

Table S5. Parmeter estimates (SE, 95% lower and upper confidence intervals) from the two most supported* superpopulation, mark-recapture models (see Table S4).

	Best model				Second best model			
Parameter**	β	SE	LCL	UCL	β	SE	LCL	UCL
Survival (ϕ)								
**Loss$_{low}$**	0.468	0.124	0.225	0.712	0.472	0.124	0.228	0.715
Fragmentation$_{low}$	0.254	0.135	-0.010	0.518	0.258	0.135	-0.006	0.522
**Matrix$_{high}$**	0.290	0.133	0.028	0.551	0.305	0.133	0.044	0.566
month$_{April}$	-1.803	0.220	-2.234	-1.371	-1.804	0.220	-2.236	-1.373
month$_{May}$	-1.261	0.254	-1.759	-0.763	-1.277	0.252	-1.771	-0.784
month$_{June}$	-1.672	0.299	-2.257	-1.087	-1.696	0.295	-2.274	-1.117
month$_{July}$	-1.883	0.153	-2.183	-1.584	-1.882	0.153	-2.182	-1.583
month$_{August}$	-2.272	0.132	-2.531	-2.014	-2.268	0.132	-2.527	-2.009
month$_{September}$	-1.835	0.113	-2.056	-1.614	-1.832	0.113	-2.053	-1.612
month$_{October}$	-0.318	0.217	-0.743	0.107	-0.316	0.217	-0.742	0.110
**Year(trend)**	-0.208	0.001	-0.210	-0.206	-0.206	0.001	-0.207	-0.204
**Loss$_{low}$ × Fragmentation$_{low}$**	-0.412	0.139	-0.684	-0.139	-0.406	0.139	-0.679	-0.134
**Loss$_{low}$ × Matrix$_{high}$**	-0.398	0.139	-0.671	-0.125	-0.409	0.139	-0.681	-0.136
Fragmentation$_{low}$ × Matrix$_{high}$	0.085	0.135	-0.179	0.349	0.072	0.135	-0.192	0.337
Probability of entry (γ)								
Loss$_{low}$	-0.187	0.243	-0.663	0.288	-0.226	0.153	-0.526	0.073
Fragmentation$_{low}$	0.094	0.269	-0.434	0.621	-0.003	0.147	-0.292	0.285
**Matrix$_{high}$**	1.219	0.319	0.595	1.844	0.579	0.148	0.290	0.869
month$_{April}$	1.138	0.438	0.280	1.996	1.116	0.438	0.258	1.974
month$_{May}$	1.831	0.373	1.100	2.562	1.809	0.373	1.077	2.540
month$_{June}$	1.752	0.398	0.972	2.531	1.780	0.396	1.003	2.557
month$_{July}$	1.853	0.355	1.157	2.550	1.858	0.356	1.161	2.555
month$_{August}$	2.444	0.346	1.765	3.122	2.447	0.346	1.768	3.126
month$_{September}$	2.398	0.349	1.713	3.082	2.401	0.350	1.716	3.086
month$_{October}$	2.622	0.372	1.892	3.352	2.627	0.373	1.896	3.357
month$_{November}$	2.554	0.413	1.744	3.363	2.558	0.413	1.747	3.368
**Year(trend)**	0.167	0.000	0.167	0.167	0.169	0.000	0.169	0.169
Loss$_{low}$ × Fragmentation$_{low}$	0.280	0.313	-0.335	0.894	NA	NA	NA	NA
Loss$_{low}$ × Matrix$_{high}$	-0.443	0.321	-1.073	0.187	NA	NA	NA	NA
**Fragmentation$_{low}$ × Matrix$_{high}$**	-0.649	0.305	-1.246	-0.052	NA	NA	NA	NA
Detection probability / probability of recapture (p)								
month$_{April}$	0.727	0.502	-0.256	1.711	0.729	0.501	-0.254	1.712
month$_{May}$	-0.044	0.339	-0.709	0.621	-0.004	0.343	-0.677	0.669
month$_{June}$	1.055	0.526	0.023	2.087	1.156	0.566	0.047	2.265
month$_{July}$	0.424	0.267	-0.099	0.948	0.413	0.267	-0.11	0.936
month$_{August}$	0.577	0.282	0.025	1.129	0.566	0.282	0.013	1.118
month$_{September}$	0.807	0.243	0.331	1.282	0.797	0.242	0.322	1.272
month$_{October}$	0.028	0.206	-0.376	0.432	0.019	0.206	-0.385	0.422
month$_{November}$	-0.283	0.199	-0.673	0.107	-0.288	0.199	-0.678	0.101
year$_{2020}$	1.072	0.204	0.671	1.473	1.062	0.205	0.661	1.462
year$_{2021}$	-0.176	0.158	-0.486	0.135	-0.196	0.158	-0.505	0.113
year$_{2022}$	-0.404	0.241	-0.876	0.068	-0.430	0.242	-0.904	0.044

*These models produced similar estimates for all parameters that were included in both models.
**significant treatment parameters based on 95% CI highlighted in bold

Table S6. Parmeter estimates (SD, 95% lower and upper credible intervals) from a hierarchical N-mixture model testing for the effects of landscape treatments, patch size, and their interactions on patch abundance.

**Parameter***	**Estimate**	**SD**	**LCL**	**UCL**	**Bayesian *P*-value****
Loss_{low}	0.095	0.578	-1.078	1.285	0.566
**$\text{Fragmentation}_{\text{low}}$**	0.713	0.546	-0.315	1.847	**0.912**
$\text{Matrix}_{\text{high}}$	0.349	0.568	-0.840	1.498	0.751
**Patch size**	0.079	0.009	0.062	0.096	**1.000**
Loss_{low} × $\text{fragmentation}_{\text{low}}$	-0.219	0.648	-1.501	1.124	0.631
Loss_{low} × $\text{matrix}_{\text{high}}$	-0.158	0.657	-1.416	1.156	0.600
$\text{Fragmentation}_{\text{low}}$ × $\text{matrix}_{\text{high}}$	0.427	0.624	-0.924	1.580	0.772
**Loss_{low} × patch size**	-0.023	0.009	-0.040	-0.005	**0.994**
**$\text{Fragmentation}_{\text{low}}$ × patch size**	-0.050	0.009	-0.068	-0.033	**1.000**
$\text{Matrix}_{\text{high}}$ × patch size	0.007	0.008	-0.010	0.022	0.806
**Loss_{low} × $\text{fragmentation}_{\text{low}}$ × patch size**	0.040	0.009	0.023	0.057	**1.000**
**Loss_{low} × $\text{matrix}_{\text{high}}$ × patch size**	-0.023	0.007	-0.037	-0.009	**0.998**
$\text{Fragmentation}_{\text{low}}$ × $\text{matrix}_{\text{high}}$ × patch size	-0.002	0.007	-0.014	0.011	0.589

*zero-inflation parameter (0.825 + 0.006 SD) and plot random effect not shown

**treatment parameters with estimated probability of effect > 0.9 in bold

Table S7. Estimates of heterogeneity in the meta-analysis focused on treatment effects.

**Test**	**I^2**	**τ**	**SE (τ)**
Amount	0.000	0.00	0.00
Fragmentation (Number of patches, NP)	13.09	0.04	0.06
Fragmentation (Isolation)	89.78	2.02	1.48
Matrix quality	96.58	6.49	3.49
Amount × fragmentation (NP)	0.000	0.00	0.00
Amount × matrix quality	74.39	0.67	0.64
Fragmentation × matrix quality	0.000	0.00	0.00

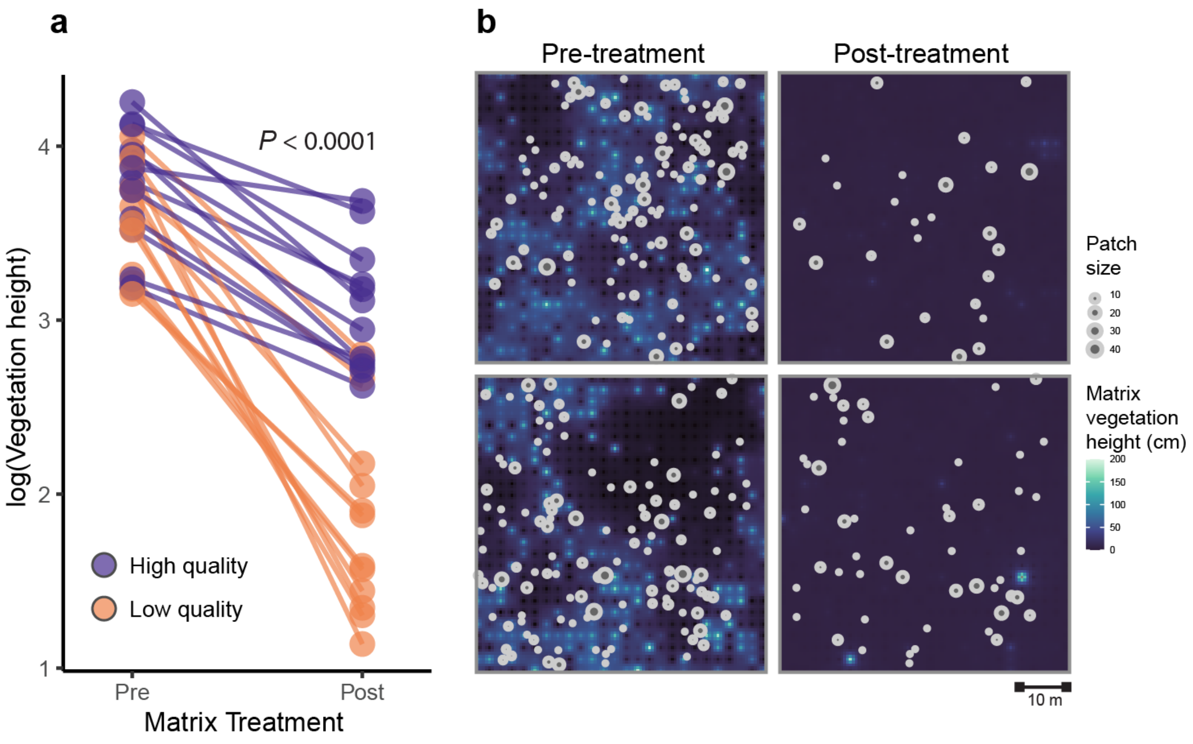

Fig. S1. Examples of habitat and matrix treatments across landscapes over time. (a) Changes in vegetation height with matrix manipulations, illustrating that treatments reduced vegetation height (N = 12 low quality; N = 12 high quality); low vegetation height has been previously estimated to increase mortality rates, such that it is a low-quality landscape matrix. (b) Shown are changes in two landscapes, where top row illustrates one landscape where a reduction in matrix quality (vegetation height), high habitat loss, and low fragmentation occurred, and bottom panel illustrates one landscape where a reduction in matrix quality, high habitat loss, and high fragmentation occurred over time. For both, the same amount of habitat was lost, but the high fragmentation treatment resulted in 2× more patches of remaining habitat (see Table S1). Vegetation height measured every 2 m across each landscape before and after treatments were applied (map created via inverse distance interpolation).

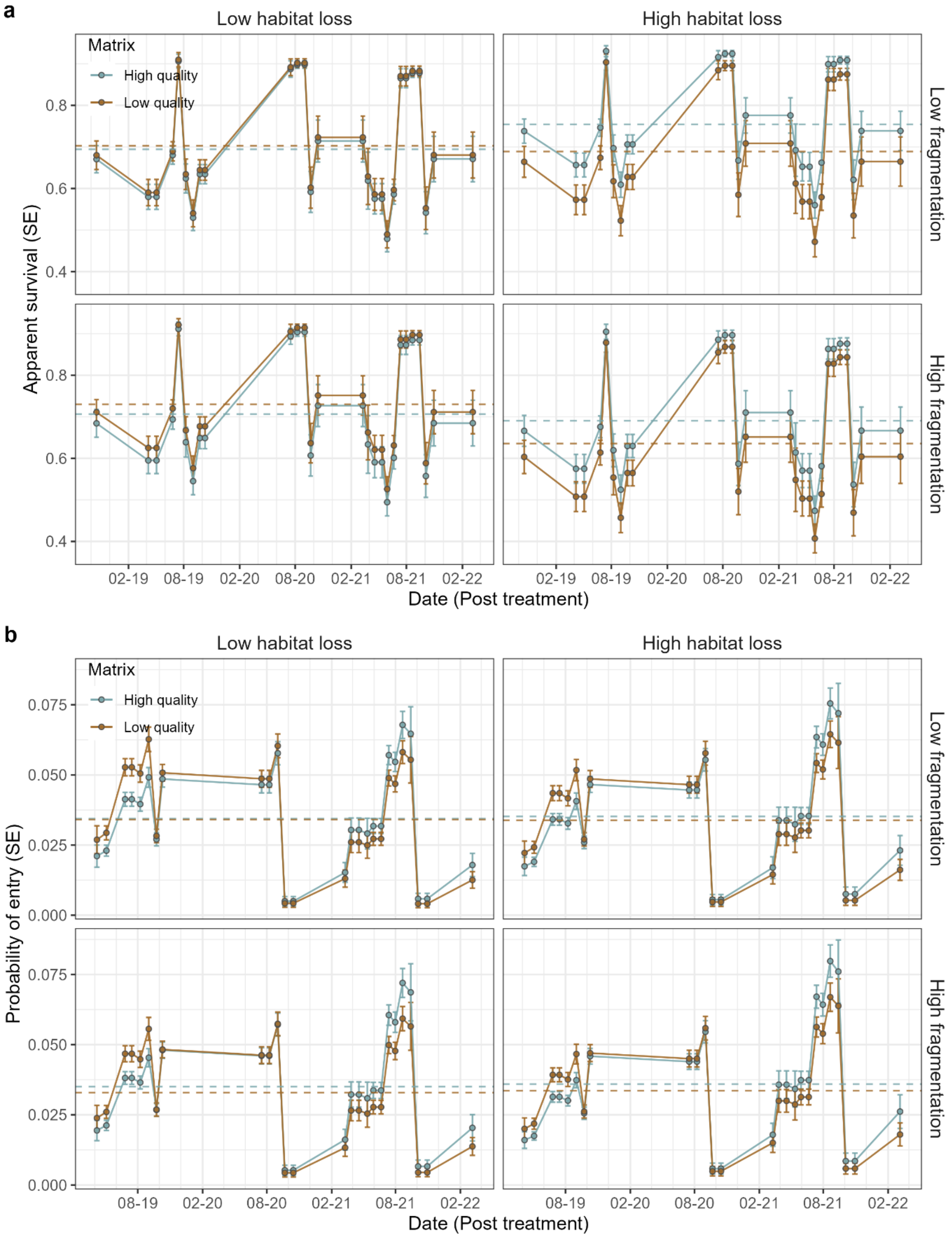

Fig. S2. Estimated survival and probability of entry (+/- SEM) over time (Date: month-year) based on the best-fitting superpopulation model. There were three landscape replicates of each treatment combination (e.g., 3 high loss, high fragmentation, low matrix quality).

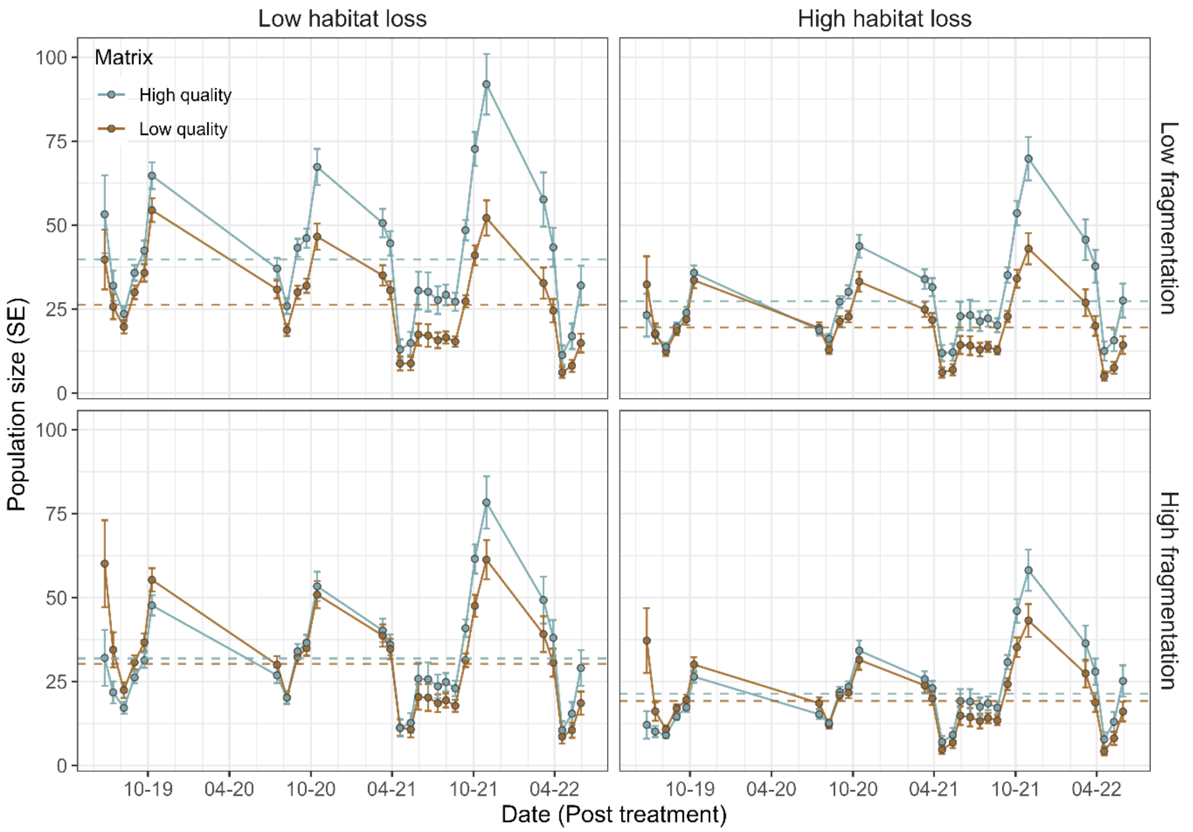

Fig. S3. Estimated population size (+/- SEM) of adults over time (Date: month-year) taken from the best-fitting superpopulation model. There were three landscape replicates of each treatment combination (e.g., 3 high loss, high fragmentation, low matrix quality).

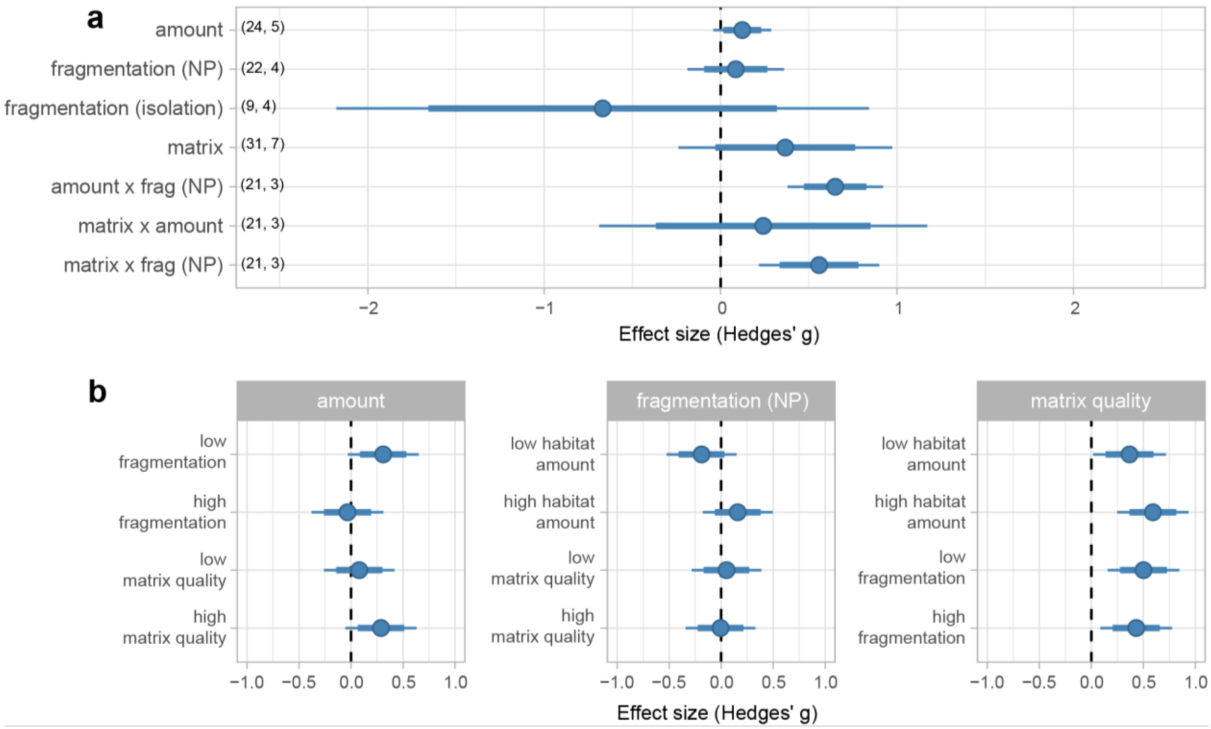

Fig. S4. Estimates from meta-analysis of experiments testing the effects of the matrix relative to habitat loss and fragmentation based on ‘focal’ and ‘pairwise’ effect sizes. Shown are estimates of (a) focal effect sizes (mean values +/- 80, 95% CI) taken from seven studies and 140 responses, and (b) pair-wise effect sizes (mean values +/- 80, 95% CI) taken from 240 responses, with each comparison including 20 effect sizes from three studies. Values in parentheses show the number of effect sizes of responses and studies, respectively.

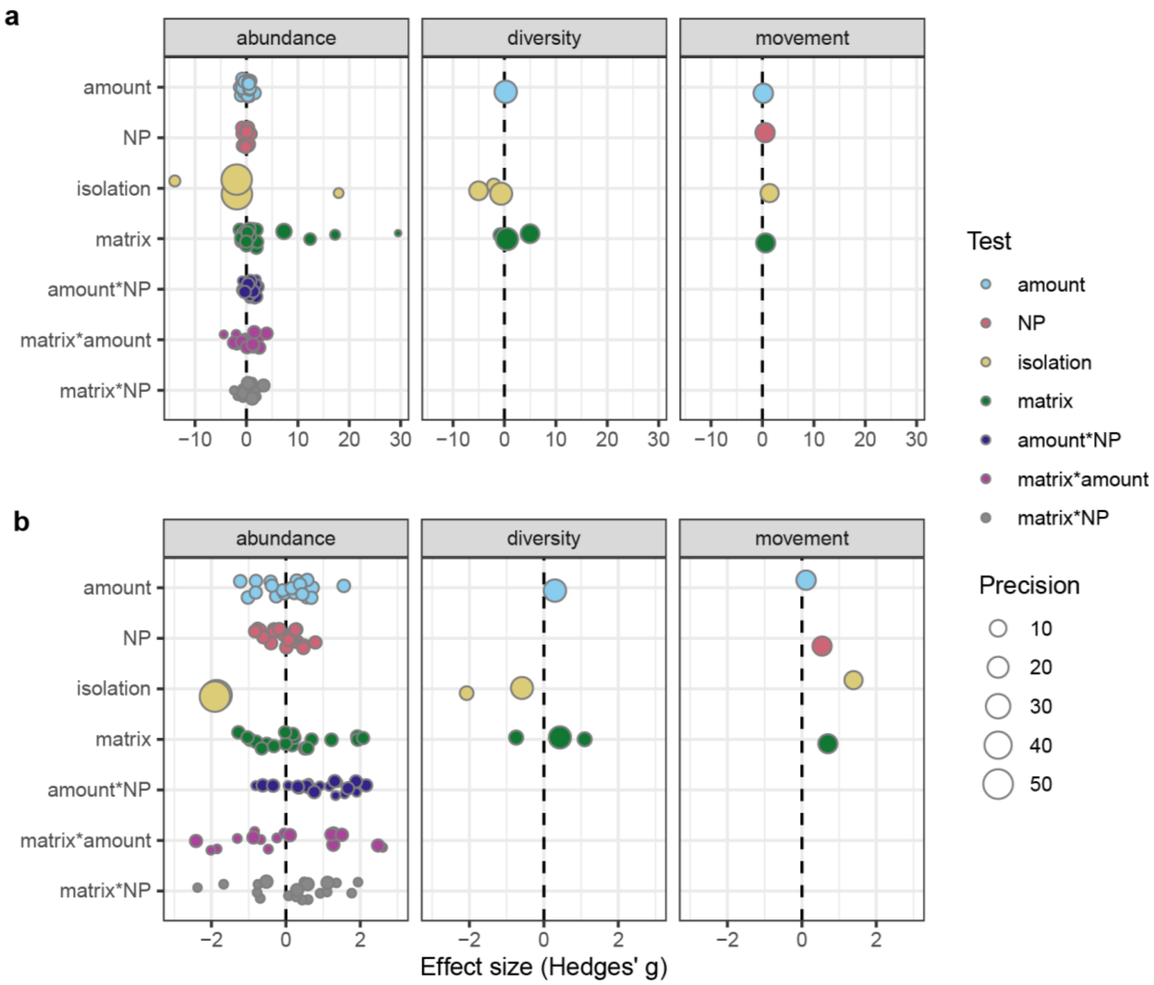

Fig. S5. Raw focal effect sizes (Hedge's g) taken from seven studies and 140 responses for (a) different types of responses. (b) Inset of effect sizes that span the -3 to 3 range.

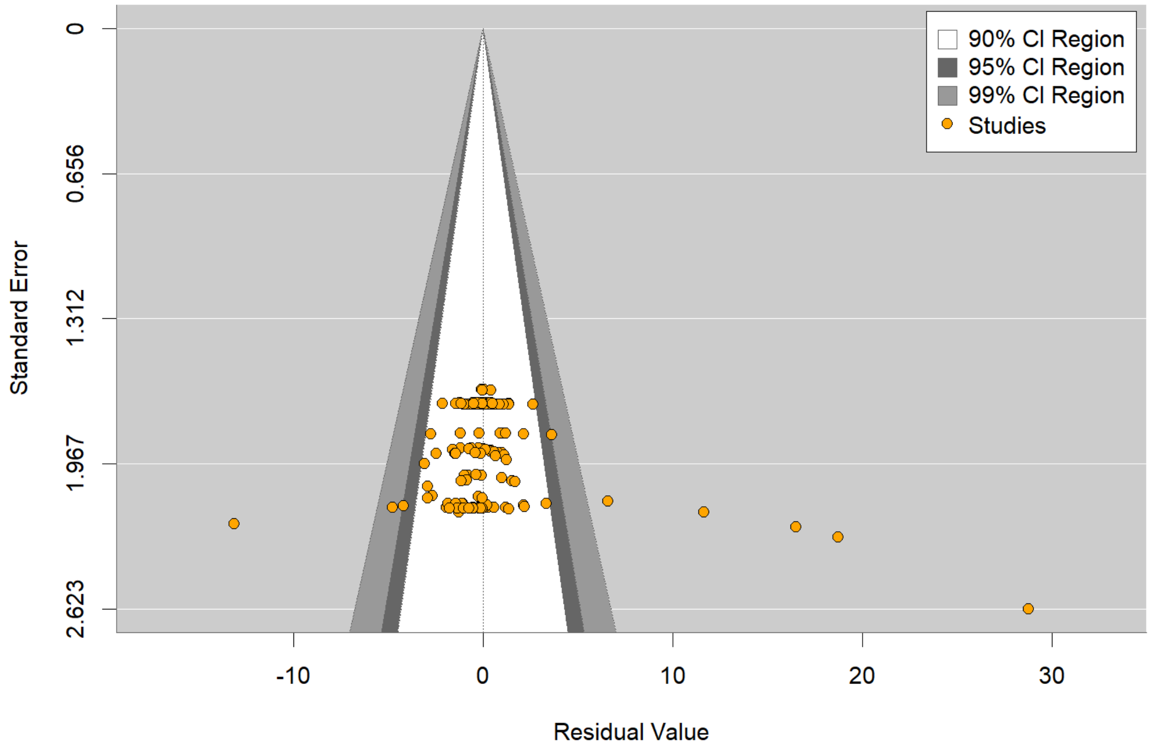

Fig. S6. Contour-enhanced funnel plot of the standard error and residuals (Hedge's g) from the full models to show the relationship between the (residual) effect sizes and precision taken from seven studies and 140 responses. The shaded areas show the approximate 90, 95 and 99% confidence regions.

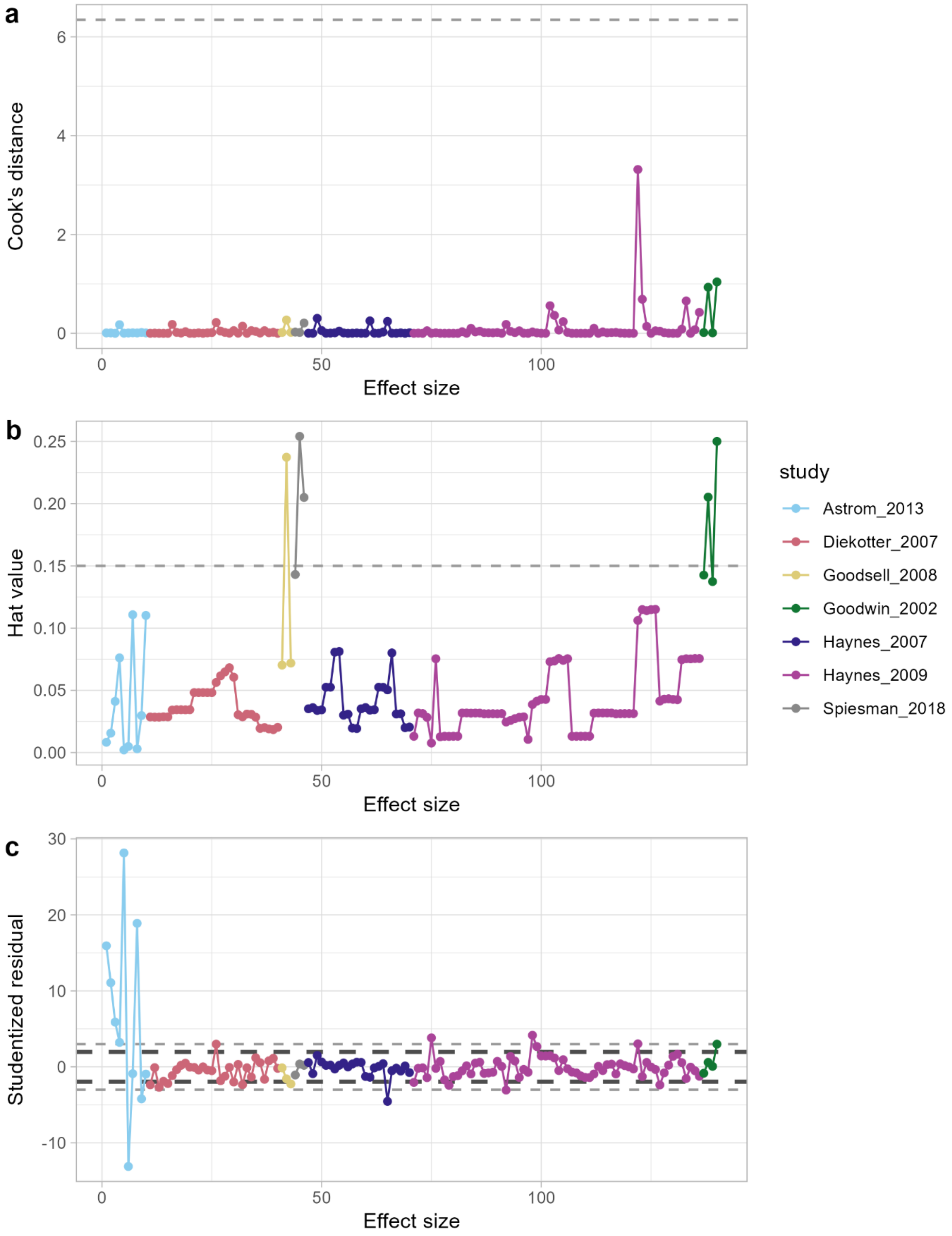

Fig. S7. Diagnostics for focal effect sizes (from Fig. S4a) for (a) Cook's distance, (b) hat values, and (c) studentized residuals. Reference lines included to show potential extreme values. For Cook's distance, we show a Chi-square threshold of 50% of the distribution to identify outliers for Cook's distance, 2 * (7/N) threshold for hat values, and 1.96 and 3 for studentized residuals.

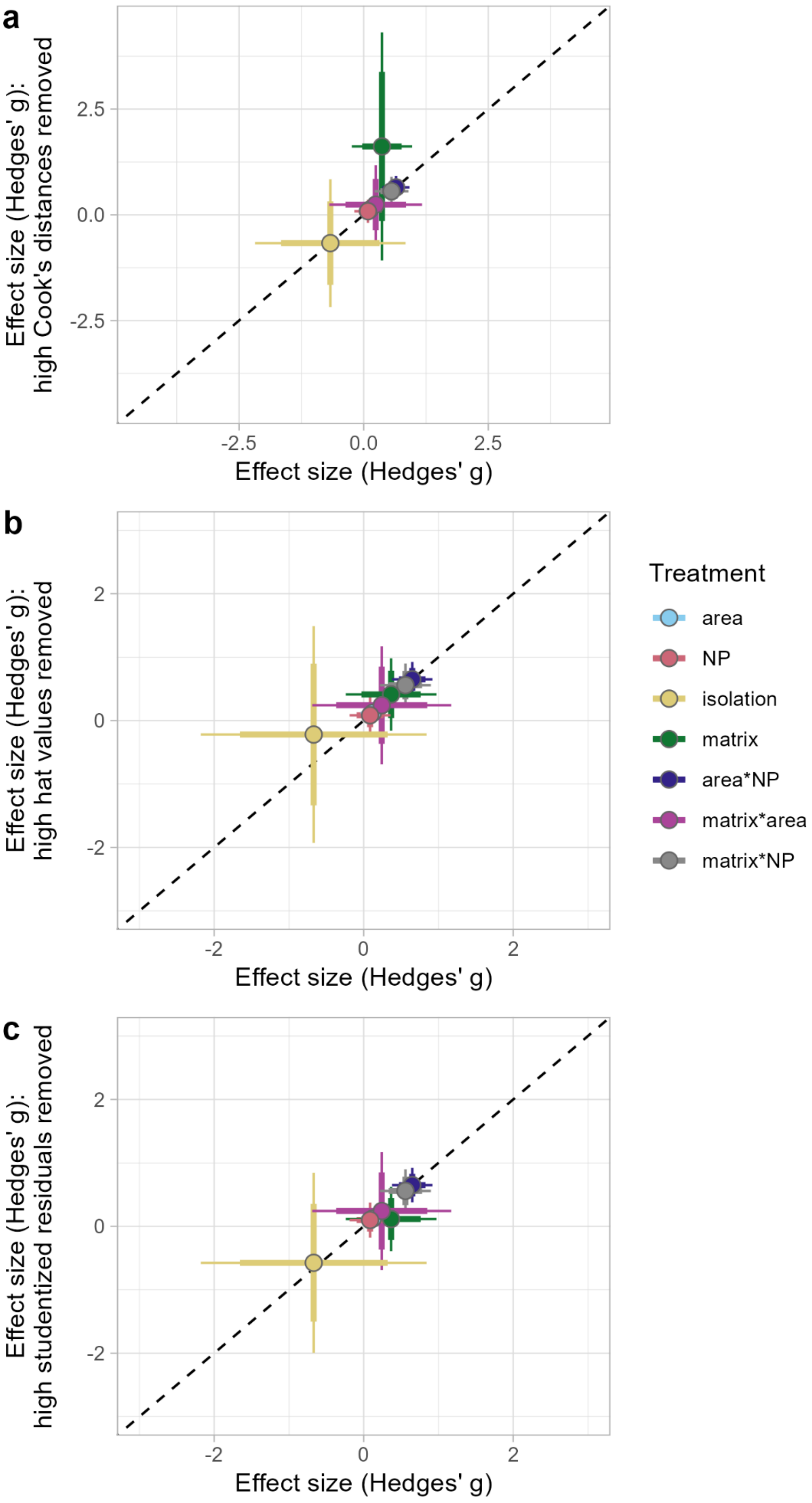

Fig. S8. Sensitivity analysis for focal effect sizes taken from seven studies and 140 responses. Shown are effect size estimates (mean values +/- 80, 95% CI) when potential outliers or influential effect sizes (identified in Fig. S7) are removed as a function of estimates when included (estimates shown in Fig S4a).

Supporting References

1 Fahrig, L. How much habitat is enough? *Biol. Conserv.* **100**, 65-74 (2001).

2 Vandermeer, J. & Carvajal, R. Metapopulation dynamics and the quality of the matrix. *Am. Nat.* **158**, 211-220 (2001). https://doi.org/10.1086/321318

3 Macartney, E. L., Lagisz, M. & Nakagawa, S. The relative benefits of environmental enrichment on learning and memory are greater when stressed: A meta-analysis of interactions in rodents. *Neuroscience and Biobehavioral Reviews* **135** (2022). https://doi.org/10.1016/j.neubiorev.2022.104554

4 Fletcher, R. J., Smith, T. A. H., Kortessis, N., Bruna, E. M. & Holt, R. D. Landscape experiments unlock relationships among habitat loss, fragmentation, and patch-size effects. *Ecology* **104**, e4037 (2023). https://doi.org/10.1002/ecy.4037

5 Astrom, J. & Part, T. Negative and matrix-dependent effects of dispersal corridors in an experimental metacommunity. *Ecology* **94**, 72-82 (2013).

6 Diekoetter, T., Haynes, K. J., Mazeffa, D. & Crist, T. O. Direct and indirect effects of habitat area and matrix composition on species interactions among flower-visiting insects. *Oikos* **116**, 1588-1598 (2007). https://doi.org/10.1111/j.2007.0030-1299.15963.x

7 Haynes, K. J. & Crist, T. O. Insect herbivory in an experimental agroecosystem: the relative importance of habitat area, fragmentation, and the matrix. *Oikos* **118**, 1477-1486 (2009). https://doi.org/10.1111/j.1600-0706.2009.17720.x

8 Haynes, K. J., Diekoetter, T. & Crist, T. O. Resource complementation and the response of an insect herbivore to habitat area and fragmentation. *Oecologia* **153**, 511-520 (2007). https://doi.org/10.1007/s00442-007-0749-4

9 Goodsell, P. J. & Connell, S. D. Complexity in the relationship between matrix composition and inter-patch distance in fragmented habitats. *Marine Biology* **154**, 117-125 (2008). https://doi.org/10.1007/s00227-008-0906-2

10 Goodwin, B. J. & Fahrig, L. How does landscape structure influence landscape connectivity? *Oikos* **99**, 552-570 (2002).

11 Spiesman, B. J., Stapper, A. P. & Inouye, B. D. Patch size, isolation, and matrix effects on biodiversity and ecosystem functioning in a landscape microcosm. *Ecosphere* **9** (2018). https://doi.org/10.1002/ecs2.2173
