# Supplementary material for: Landscape quality drives ecological responses to habitat loss and fragmentation
Source: Nat Ecol Evol. 2026 Jun 17;10(7):1265–72. doi: 10.1038/s41559-026-03095-1 (PMC13346112; doi:10.1038/s41559-026-03095-1)
Supplement: Supplementary file 2 — Reporting Summary [file 41559_2026_3095_MOESM2_ESM.pdf]

nature portfolio

Corresponding author(s): Robert J. Fletcher

Last updated by author(s): Apr 9, 2026

Reporting Summary

Nature Portfolio wishes to improve the reproducibility of the work that we publish. This form provides structure for consistency and transparency in reporting. For further information on Nature Portfolio policies, see our Editorial Policies and the Editorial Policy Checklist.

Statistics

For all statistical analyses, confirm that the following items are present in the figure legend, table legend, main text, or Methods section.

n/a	Confirmed	
☐	☒	The exact sample size (n) for each experimental group/condition, given as a discrete number and unit of measurement
☐	☒	A statement on whether measurements were taken from distinct samples or whether the same sample was measured repeatedly
☐	☒	The statistical test(s) used AND whether they are one- or two-sided<br>*Only common tests should be described solely by name; describe more complex techniques in the Methods section.*
☐	☒	A description of all covariates tested
☐	☒	A description of any assumptions or corrections, such as tests of normality and adjustment for multiple comparisons
☐	☒	A full description of the statistical parameters including central tendency (e.g. means) or other basic estimates (e.g. regression coefficient) AND variation (e.g. standard deviation) or associated estimates of uncertainty (e.g. confidence intervals)
☐	☒	For null hypothesis testing, the test statistic (e.g. F, t, r) with confidence intervals, effect sizes, degrees of freedom and P value noted<br>*Give P values as exact values whenever suitable.*
☐	☒	For Bayesian analysis, information on the choice of priors and Markov chain Monte Carlo settings
☐	☒	For hierarchical and complex designs, identification of the appropriate level for tests and full reporting of outcomes
☒	☐	Estimates of effect sizes (e.g. Cohen's d, Pearson's r), indicating how they were calculated

Our web collection on statistics for biologists contains articles on many of the points above.

Software and code

Policy information about availability of computer code

Data collection	All data used in this manuscript can be found at Figshare: 10.6084/m9.figshare.30508496.
Data analysis	All analyses were conducted in R 4.4.1 and Jags 4.3.1. Packages used in R include: tidyverse (2.0), lubridate (1.9.4), RMark (3.0), emmeans (1.10.7), metafor (4.8-0), broom (1.0.7), and jagsUI (1.6.2). Code used for conducting analyses can be found at Figshare: 10.6084/m9.figshare.30508496.

For manuscripts utilizing custom algorithms or software that are central to the research but not yet described in published literature, software must be made available to editors and reviewers. We strongly encourage code deposition in a community repository (e.g. GitHub). See the Nature Portfolio guidelines for submitting code & software for further information.

Data

Policy information about availability of data

All manuscripts must include a data availability statement. This statement should provide the following information, where applicable:

- Accession codes, unique identifiers, or web links for publicly available datasets
- A description of any restrictions on data availability
- For clinical datasets or third party data, please ensure that the statement adheres to our policy

All data and code used in this manuscript can be found at Figshare: 10.6084/m9.figshare.30508496.

Research involving human participants, their data, or biological material

Policy information about studies with human participants or human data. See also policy information about sex, gender (identity/presentation), and sexual orientation and race, ethnicity and racism.

Reporting on sex and gender	NA
Reporting on race, ethnicity, or other socially relevant groupings	NA
Population characteristics	NA
Recruitment	NA
Ethics oversight	NA

Note that full information on the approval of the study protocol must also be provided in the manuscript.

Field-specific reporting

Please select the one below that is the best fit for your research. If you are not sure, read the appropriate sections before making your selection.

☒ Life sciences ☐ Behavioural & social sciences ☐ Ecological, evolutionary & environmental sciences

For a reference copy of the document with all sections, see nature.com/documents/nr-reporting-summary-flat.pdf

Life sciences study design

All studies must disclose on these points even when the disclosure is negative.

Sample size	The number of landscapes considered was constrained based on the study area size. We maximized the total number of possible landscapes within the study area, maintaining a minimum separation between landscape units. The number of patches within landscapes was determined based on prior sampling in study area to reflect natural densities of patches.
Data exclusions	The only data that were excluded were resighting data of marked individuals where the mark had not been applied (and so was an error in resighting).
Replication	All attempts at replication were successful. Each landscape treatment combination was replicated three times, for a total of 24 treatment landscapes and 3 control landscapes.
Randomization	Treatments were allocated randomly within blocks.
Blinding	Investigators were not blinded to treatments during data collection and analysis. Blinding during data collection was not possible because investigators could ascertain the number of patches and the landscape quality while collecting data in the field.

Reporting for specific materials, systems and methods

We require information from authors about some types of materials, experimental systems and methods used in many studies. Here, indicate whether each material, system or method listed is relevant to your study. If you are not sure if a list item applies to your research, read the appropriate section before selecting a response.

Materials & experimental systems

n/a	Involved in the study	
☒	☐	Antibodies
☒	☐	Eukaryotic cell lines
☒	☐	Palaeontology and archaeology
☐	☒	Animals and other organisms
☒	☐	Clinical data
☒	☐	Dual use research of concern
☐	☒	Plants

Methods

n/a	Involved in the study	
☒	☐	ChIP-seq
☒	☐	Flow cytometry
☒	☐	MRI-based neuroimaging

Animals and other research organisms

Policy information about studies involving animals; ARRIVE guidelines recommended for reporting animal research, and Sex and Gender in Research

Laboratory animals	NA
Wild animals	Chelinidea vittiger (nymphs and adults) was counted in the field during each survey. Unmarked adults were gently captured by hand and placed in a small tube to transport to a marking station (adjact to each plot). Each individual was handled for approximately 5 minutes for marking. Marking included adhering a small card-stock (4 x 6 mm) containing three-letter mark typed on the card-stock. This was placed on the protonum of C. vittiger. Greenhouse experiments previously showed that this marking strategy has no impacts on survival. Immediately after marking, individual C. vittiger were returned to the location of capture.
Reporting on sex	Sex was determined for adult C. vittiger using visual attributes. Because this was not possible for all adults, we did not analyze effects of sex in this article.
Field-collected samples	NA
Ethics oversight	No ethical approval was required because study taxon was insects (which was not covered by the Animal Care Committee).

Note that full information on the approval of the study protocol must also be provided in the manuscript.

Dual use research of concern

Policy information about dual use research of concern

Hazards

Could the accidental, deliberate or reckless misuse of agents or technologies generated in the work, or the application of information presented in the manuscript, pose a threat to:

No	Yes	
☒	☐	Public health
☒	☐	National security
☒	☐	Crops and/or livestock
☒	☐	Ecosystems
☒	☐	Any other significant area

Experiments of concern

Does the work involve any of these experiments of concern:

No	Yes	
☒	☐	Demonstrate how to render a vaccine ineffective
☒	☐	Confer resistance to therapeutically useful antibiotics or antiviral agents
☒	☐	Enhance the virulence of a pathogen or render a nonpathogen virulent
☒	☐	Increase transmissibility of a pathogen
☒	☐	Alter the host range of a pathogen
☒	☐	Enable evasion of diagnostic/detection modalities
☒	☐	Enable the weaponization of a biological agent or toxin
☒	☐	Any other potentially harmful combination of experiments and agents

Plants

Seed stocks	All plants (Opuntia mesacantha) used in the experiment came from the study area where research was conducted.
Novel plant genotypes	NA
Authentication	NA
