# Supplementary material for: Landscape quality drives ecological responses to habitat loss and fragmentation
Source: Nat Ecol Evol. 2026 Jun 17;10(7):1265–72. doi: 10.1038/s41559-026-03095-1 (PMC13346112; doi:10.1038/s41559-026-03095-1)
Supplement: Supplementary file 3 — Peer Review File [file 41559_2026_3095_MOESM3_ESM.pdf]

nature portfolio

Peer Review File

Landscape quality drives ecological responses to habitat loss and fragmentation

Corresponding Author: Professor Robert Fletcher

Version 0:

Decision Letter:

3rd December 2025

*Please ensure you delete the link to your author homepage in this e-mail if you wish to forward it to your co-authors.

Dear Rob,

Your manuscript entitled "Landscape quality drives ecological responses to habitat loss and fragmentation" has now been evaluated by three reviewers. As you will see from the reports below, the reviewers find your work of considerable potential interest but have raised quite substantial concerns to address. In light of their feedback, we would like to see a revised manuscript along with a response to all the reviewers' comments.

Regarding the meta-analysis, we encourage you to follow Reviewer 1's suggestions to improve its methodological rigour and transparency, but given the extremely small number of available source studies we agree with Reviewer 2's request to treat its results as qualitative patterns rather than quantitative benchmarks. Perhaps it could even be relegated to the Supplementary Information with appropriate mentions in the main text. Regardless, our main editorial interest is in the experimental component, even after toning down claims on the generality of the findings as requested by the reviewers.

In addition, please ensure that the novelty relative to your previous works on this system (especially references 34 and 36) is better articulated, to help the readers follow how the conceptual framing builds from earlier findings and what is genuinely new here.

We are committed to providing a fair and constructive peer-review process. Please do not hesitate to contact us if there are specific requests from the reviewers that you believe are technically impossible or unlikely to yield a meaningful outcome.

When revising your manuscript:

* Please provide the manuscript in Microsoft Word format and highlight all changes in the same file or a copy.

* Include a “Response to reviewers” document detailing, point-by-point, how you addressed each referee comment. If no action was taken to address a point, you must provide a compelling argument. This response will be sent back to the referees along with the revised manuscript.

* If you have not done so already we suggest that you begin to revise your manuscript so that it conforms to our Article format instructions at http://www.nature.com/natecolevol/info/final-submission. Refer also to any guidelines provided in this letter.

* Include a revised version of any required reporting checklist. It will be available to referees (and, potentially, statisticians) to aid in their evaluation if the manuscript goes back for peer review. A revised checklist is essential for re-review of the paper.

If you wish to submit a suitably revised manuscript, we would hope to receive it within three months. If you cannot send it within this time, please let us know. We will be happy to consider your revision so long as nothing similar has been accepted for publication at Nature Ecology & Evolution or published elsewhere. Please use the link below to submit a revised paper:

Link Redacted

Note: This URL links to your confidential home page and associated information about manuscripts you may have submitted, or that you are reviewing for us. If you wish to forward this email to co-authors, please delete the link to your homepage.

Please do not hesitate to contact me if you have any questions or would like to discuss the required revisions further.

[redacted]

Reviewers' comments:

Reviewer #1 (Remarks to the Author):

This is a great study investigating the effects of habitat loss, fragmentation and matrix quality on biodiversity across scales. The authors performed an excellent experiment manipulating both habitat amount, fragmentation and matrix quality to test their relative contribution to population size. In addition to that, the authors performed a meta-analysis to aggregate experiments comparing / manipulating the same drivers. Surprisingly, the authors found very few experiments comparing these three landscape drivers of biodiversity change, but their results indicate that matrix quality is an essential variable to fully understand how habitat loss and fragmentation affect population dynamics. I consider these results needed to improve our ability to address conservation challenges in how to prioritize biodiversity protection, design reserves, and ultimately to mitigate the ongoing climate crisis and its effects on natural ecosystems. However, I do have some concerns, especially with the way the authors reported the meta-analyses. I believe there are several aspects of the meta-analysis that can be better explained. Below I provide a non-exhaustive list that can help authors improve it:

o How did you estimate heterogeneity? You mentioned QM statistics, but there is no information about between-study variance, how it varies across random effects. This is partially performed in the code, but it deserves more explanation both in the text and code.

o How did you assess publication bias? The classic approaches (e.g., funnel plots, trim-and-fill, and others) were ignored, or I did not find them in the supp material (my bad if that's the case).

o How did you test whether some studies affected your main results? Sensitivity analyses and influence diagnostics? How did you check for outliers? Because this is not clearly explained, there is some information / decisions that are challenging to understand how you did that. For example, Figure 3 says "Positive effect of isolation driven by one experiment on corridors", but there is nothing explaining how you did that and how this affected the analyses.

o Better define the selected response types and how they were treated in the model. Figure S5 shows you used movement, function, diversity and abundance, but how these variables are comparable across studies is not clearly articulated. For example, how did you compare 'function' across studies? How did you compare abundance across different taxonomic groups such as bacteria, arthropods and microarthropods? Even within arthropods, comparing, let's say, spiders with ants, the difference in abundance can be huge. Moreover, it looks like in the model of Figure 3 you are using all effect sizes, which means you are combining studies that used movement with diversity? I'm afraid this is comparing apples with oranges and how you considered these differences is also not clearly explained.

o I also believe you must explain how this meta-analysis will be used to test Figure 1 predictions. In fact, it is hard to read the results from Figure 3 and automatically connect them with Figure 1. For instance, if fragmentation (isolation) has a negative effect, which prediction is it testing as you are not controlling for habitat amount / quality? In my view, this is because you defined the landscape elements, matrix, habitat area, habitat fragmentation and pairwise interactions without showing how the isolated and interacting effects connect with the predictions.

o An additional comment about the landscape elements is the inability to understand how the original studies defined 'habitat quality'. While habitat amount/area and fragmentation (kind of, although people clearly define it in multiple ways) are more obvious, habitat quality is more challenging. Is this the same binary (high vs. low) definition as you defined in the experiment? In the text you described it as "calculated matrix effect sizes as the difference between the sum of the mean responses in all experimental landscapes with one type of matrix and the sum of mean responses in landscapes with the alternative matrix type", but this is not clear enough.

Minor comments

- Introduction: As you briefly describe the experiment at the end of the introduction, it is important to describe some relevant information about the studies compiled in the meta-analysis.

- Discussion: In the sentence "Both our landscape experiment and meta-analysis emphasize that the quality of the surrounding landscape matrix can have large effects on populations and communities" (L162) it's not clear how your experiment allows us to understand effects on communities. I believe the only response type allowing conclusions about communities is the diversity component from the meta-analysis. Importantly, it looks like there are three studies that use diversity (Figure S5). I believe changing some points I raised about the meta-analysis would help readers to understand the interpretations of the meta-analysis.

- Conceptual figure: I believe there are some confusing aspects to this figure that could be improved. Figure 1a for example

describes the effect of fragmentation vs. quality of a given response. However, the text is using fragmentation AND loss. I'm not sure if you expect that their effects and interaction with quality are the same, or if you expect that their combined effects will interact with quality to determine its response. Even if the answer is one or another, I believe this is difficult to follow and deserves a better explanation. The introduction covers these differences, but the conceptual figure is somewhat making these predictions a bit hard to understand.

Reviewer #1 (Remarks on code availability):

I did review the code and it looks ok. However, I expect major changes in the meta-analyses code, as I suggested in the comments for the authors.

Reviewer #2 (Remarks to the Author):

This manuscript presents a large-scale, long-term experiment manipulating habitat loss, fragmentation, and matrix quality in a factorial design, complemented by a meta-analysis of all existing experiments that concurrently manipulate habitat and matrix components. The authors use a focal host–plant specialist (Chelinidea vittiger) in a semi-natural system and integrate demographic modeling (POPAN) with patch-level abundance models to examine multi-scale population responses. The central claim is that landscape matrix quality can have effects on demographic rates and population size comparable to, or greater than, habitat loss and fragmentation, and that these effects are strongest when loss and fragmentation are low.
The topic is timely and relevant, and the authors address an important gap in the experimental literature. The experiment is ambitious in scope and represents one of the most comprehensive landscape manipulations available. The manuscript is clearly written and well framed in the context of ongoing debates in landscape ecology.
The authors have a strong track record working with this system, and this manuscript builds on several of their previous publications manipulating habitat configuration, studying dispersal, and examining matrix effects. While much of the conceptual framing and system-specific insights extend earlier work, this study offers clear empirical advances: (i) the simultaneous manipulation of habitat loss, fragmentation, and matrix quality in a fully factorial design; (ii) long-term demographic inference across 12 generations; and (iii) the first meta-analysis exclusively integrating experiments that manipulate both habitat and matrix characteristics. These represent incremental but meaningful contributions beyond the authors' prior research.
Weaknesses
1. Generality is limited to a single specialist insect in a highly specific habitat-matrix system.
2. Some figures referenced (notably Fig. 2d–e) did not appear in the version reviewed.
3. Interpretation of interactions among loss, fragmentation, patch size, and matrix quality is sometimes qualitative; effect sizes or partial dependence plots would improve clarity.
4. Meta-analysis limitations: only five unique experiments are available, and several effect-size categories are dominated by single studies. This restricts statistical robustness and weakens quantitative generalizations.
5. Heterogeneity across studies in how "fragmentation" and "matrix quality" are defined complicates inference.
Statistical comments regarding the meta-analysis:
The meta-analysis is a useful addition but must be interpreted cautiously. The low number of studies, substantial heterogeneity in experimental manipulations, non-independence among multiple effect sizes per article, and the dominance of isolated experiments for some landscape elements all constrain the strength of the conclusions. It is surprising that confidence intervals shown in Fig. 3 for each moderator are identical while their sample sizes are different (especially for Fragmentation (Isolation) with a sample size of 9). While the multilevel meta-analytic framework is appropriate, the resulting estimates should be treated as qualitative patterns rather than firm quantitative benchmarks. The authors should temper claims about generality and acknowledge these statistical limitations more explicitly.

Reviewer #3 (Remarks to the Author):

This is a highly valuable study. In my opinion, it is unparalleled in its value for understanding the independent and interacting effects of habitat loss, fragmentation, and matrix quality on population abundance. Too often, these factors are either confounded or one (usually matrix quality) is simply ignored. Not only were these factors manipulated independently, but this was done at an appropriately large spatial scale. In addition, the authors were able to explain some of the underlying demographic mechanisms.

My main concern, which could probably be addressed easily, involves the topic of land sharing versus land sparing seemed. The authors did not clearly articulate what these terms, which may be unfamiliar to many ecologists, mean or how their study is relevant to these strategies for balancing food production with conservation. I found the concluding paragraph of the Discussion, which focuses on this topic, difficult to understand. The meaning of the first sentence in that paragraph is particularly unclear. To me, the topic does not seem directly relevant. Second, when interpreting the results of a statistical model and the interaction term for two variables is significant, it is fruitless to interpret what the results mean for the main effects of the variables involved in the interaction.

Version 1:

Decision Letter:

26th March 2026

Dear Dr. Fletcher,

Thank you for submitting your revised manuscript "Landscape quality drives ecological responses to habitat loss and fragmentation" (NATECOLEVOL-25103811A). Based on Reviewer 1's comments and our assessment of the revisions made in response to the other reviewers, we will be happy in principle to publish it in Nature Ecology & Evolution, pending some final revisions to satisfy the reviewers' final requests and to comply with our editorial and formatting guidelines.

We are now performing detailed checks on your paper and will send you a checklist detailing our editorial and formatting requirements in about a week. Please do not upload the final materials and make any revisions until you receive this additional information from us.

Thank you again for your interest in Nature Ecology & Evolution. Please do not hesitate to contact me if you have any questions.

[redacted]

Reviewer #1 (Remarks to the Author):

This paper will be a great contribution to the field. I still think, however, the meta-analysis is not changing (or reinforcing) the main results supported by the experiment. In fact, the current version is overselling the review/meta-analysis generality. For example, "We reviewed 2,221 articles..." This is actually the number of screened papers, and the results were extracted from 7 papers only.

Reviewer #1 (Remarks on code availability):

The code works great and I tested it. No major problems. Easy to go through.

Open Access This Peer Review File is licensed under a Creative Commons Attribution 4.0 International License, which permits use, sharing, adaptation, distribution and reproduction in any medium or format, as long as you give appropriate credit to the original author(s) and the source, provide a link to the Creative Commons license, and indicate if changes were made.
In cases where reviewers are anonymous, credit should be given to 'Anonymous Referee' and the source.
The images or other third party material in this Peer Review File are included in the article's Creative Commons license, unless indicated otherwise in a credit line to the material. If material is not included in the article's Creative Commons license and your intended use is not permitted by statutory regulation or exceeds the permitted use, you will need to obtain

permission directly from the copyright holder.
To view a copy of this license, visit https://creativecommons.org/licenses/by/4.0/

Reviewer #1 (Remarks to the Author):

This is a great study investigating the effects of habitat loss, fragmentation and matrix quality on biodiversity across scales. The authors performed an excellent experiment manipulating both habitat amount, fragmentation and matrix quality to test their relative contribution to population size. In addition to that, the authors performed a meta-analysis to aggregate experiments comparing / manipulating the same drivers. Surprisingly, the authors found very few experiments comparing these three landscape drivers of biodiversity change, but their results indicate that matrix quality is an essential variable to fully understand how habitat loss and fragmentation affect population dynamics. I consider these results needed to improve our ability to address conservation challenges in how to prioritize biodiversity protection, design reserves, and ultimately to mitigate the ongoing climate crisis and its effects on natural ecosystems. However, I do have some concerns, especially with the way the authors reported the meta-analyses. I believe there are several aspects of the meta-analysis that can be better explained. Below I provide a non-exhaustive list that can help authors improve it:

o How did you estimate heterogeneity? You mentioned QM statistics, but there is no information about between-study variance, how it varies across random effects. This is partially performed in the code, but it deserves more explanation both in the text and code.

>>>Response. Thank you for this question. We had explored heterogeneity in the original analysis by calculating the I^2 statistic to address this issue, but we realize that we did not report on it in any detail.

In the revised manuscript, we include two types of analyses for estimating heterogeneity. First, we report the I2 statistic for between-study variance when no moderators were considered. Second, We calculate both the I^2 statistic and tau for estimating heterogeneity in the meta-regression we focus on for inferences of treatment effects. In this way, we report both I^2 and tau in Table S7 of the Supporting Information.

o How did you assess publication bias? The classic approaches (e.g., funnel plots, trim-and-fill, and others) were ignored, or I did not find them in the supp material (my bad if that's the case).

>>>Response. Given that we only included experiments that manipulated both habitat and the matrix, we expect that publication bias is less prevalent than in situations where only one factor is considered. We had initially explored using funnel plots but did not include them in the original submission.

In the revised manuscript, we add a contour-enhanced funnel plot (Peters et al. 2008) to the Supporting Information (Figure S4 of revised manuscript). This plot does not appear to show potential publication bias.

o How did you test whether some studies affected your main results? Sensitivity analyses and

influence diagnostics? How did you check for outliers? Because this is not clearly explained, there is some information / decisions that are challenging to understand how you did that. For example, Figure 3 says “Positive effect of isolation driven by one experiment on corridors”, but there is nothing explaining how you did that and how this affected the analyses.

>>>Response. In the original submission, we were concerned with the limited sample size in the meta-analysis, particularly for the isolation test which had the fewest number of studies. In that statement, we had re-run the analysis without the study included, which altered results (which was the basis of that statement).

In the revised manuscript, we include a more formal sensitivity and diagnostic analysis. First, we calculated Cook’s distance, hat values, and studentized residuals (Fig. S5 of the revised manuscript). Then we used common thresholds for each to identify potential influential effect sizes (those that appeared to be outliers or with high leverage; Viechtbauer and Cheung 2010). We re-ran the meta-analysis removing those points and compared estimates to those from the full data set (Fig. S6). In general, there were some influential effect sizes, particularly from one study that we estimated large effect sizes (Astrom and Part 2013). However, this study was also the experiment with greatest replication (Table S1), such that estimates from that study were very precise in comparison to other studies. When we remove effect sizes identified as influential from these diagnostics, estimates were largely similar (Fig. S6 of revised manuscript).

o Better define the selected response types and how they were treated in the model. Figure S5 shows you used movement, function, diversity and abundance, but how these variables are comparable across studies is not clearly articulated. For example, how did you compare ‘function’ across studies? How did you compare abundance across different taxonomic groups such as bacteria, arthropods and microarthropods? Even within arthropods, comparing, let’s say, spiders with ants, the difference in abundance can be huge. Moreover, it looks like in the model of Figure 3 you are using all effect sizes, which means you are combining studies that used movement with diversity? I’m afraid this is comparing apples with oranges and how you considered these differences is also not clearly explained.

>>>Response. Regarding the different response variable categories, we estimated effect sizes for measures of abundance and diversity such that a positive effect indicated that the given measure was greater in landscapes with higher quality matrix, more habitat, or less fragmentation. We defined effect sizes as negative when abundance or diversity metrics were lower in these “better” landscapes. For movement, only one metric was used in the selected studies, patch immigration (mean number of immigrants per patch), and we estimated these effect sizes as positive when immigration was higher in the “better” landscape. Given this comment, we decided to remove effect sizes on ecological function because there were only two direct measurements of function (herbivory and decomposition) and it was not clear how to best assume directionality of effect sizes for herbivory. In terms of abundance comparisons across taxa, we assume that by using Hedge’s g, which is in units of standard deviations, comparisons across taxa are appropriate.

In the revised manuscript, we added more text that better describes this process, along with the rationale for using Hedge's g and its relevance across taxa on Lines 354-358, 379-384.

o I also believe you must explain how this meta-analysis will be used to test Figure 1 predictions. In fact, it is hard to read the results from Figure 3 and automatically connect them with Figure 1. For instance, if fragmentation (isolation) has a negative effect, which prediction is it testing as you are not controlling for habitat amount / quality? In my view, this is because you defined the landscape elements, matrix, habitat area, habitat fragmentation and pairwise interactions without showing how the isolated and interacting effects connect with the predictions.

>>>Response. Thank you for this comment. Based on this issue, we re-visited meta-analysis effect size estimation and extracted pair-wise effect sizes in addition to the total effect sizes that were previously reported, following the approach of Gurvetich et al. (2000) and Macartney et al. (2022). These pair-wise effect sizes are much easier to interpret and link more closely to Figure 1 and are reported in Fig. 3b.

o An additional comment about the landscape elements is the inability to understand how the original studies defined 'habitat quality'. While habitat amount/area and fragmentation (kind of, although people clearly define it in multiple ways) are more obvious, habitat quality is more challenging. Is this the same binary (high vs. low) definition as you defined in the experiment? In the text you described it as "calculated matrix effect sizes as the difference between the sum of the mean responses in all experimental landscapes with one type of matrix and the sum of mean responses in landscapes with the alternative matrix type", but this is not clear enough.

>>>Response. We used the authors' descriptions to code 'matrix quality' in the meta-analysis whenever possible; however, when this was not possible, we selected the matrix type with greatest contrast to habitat as 'low quality'. For example, Astrom and Part (2013) state, "Matrix quality was manipulated by contour-sawing the plywood sheets at the border of the moss fragments, leading to the moss habitats being directly surrounded either by gravel or by the plywood surface. The gravel matrix was expected to be a significantly worse matrix habitat, in terms of both permeability and microclimate, because the gravel bed constitutes a considerable topography relative to the species involved and can reach high temperatures during summer days due to solar radiation". So for this experiment, we considered gravel matrix as lower quality. In general, across experiments, low-quality matrix was that with greatest contrast to habitat.

In the revised manuscript, we add a statement that describes this approach on Lines 368-371 and some Discussion of this issues on lines 186-189.

Minor comments

- Introduction: As you briefly describe the experiment at the end of the introduction, it is

important to describe some relevant information about the studies compiled in the meta-analysis.

>>>Response. We added a brief overview at the end of the Introduction on Lines 93-95 of the revised manuscript.

- Discussion: In the sentence "Both our landscape experiment and meta-analysis emphasize that the quality of the surrounding landscape matrix can have large effects on populations and communities" (L162) it's not clear how your experiment allows us to understand effects on communities. I believe the only response type allowing conclusions about communities is the diversity component from the meta-analysis. Importantly, it looks like there are three studies that use diversity (Figure S5). I believe changing some points I raised about the meta-analysis would help readers to understand the interpretations of the meta-analysis.

>>>Response. Thank you for this comment. We had initially used 'communities' referring to the meta-analysis and both effect sizes based on diversity and function (most of the 'function' responses reflected species interactions that contribute to function).

In the revised manuscript, we simply removed 'populations and communities' in this sentence but we also further elaborate on this issue and the limited community responses that were tracked during experiments on Lines 191-196.

- Conceptual figure: I believe there are some confusing aspects to this figure that could be improved. Figure 1a for example describes the effect of fragmentation vs. quality of a given response. However, the text is using fragmentation AND loss. I'm not sure if you expect that their effects and interaction with quality are the same, or if you expect that their combined effects will interact with quality to determine its response. Even if the answer is one or another, I believe this is difficult to follow and deserves a better explanation. The introduction covers these differences, but the conceptual figure is somewhat making these predictions a bit hard to understand.

>>>Response. Thank you for this response. In general, most models and theory make similar predictions for how matrix quality may interact with both habitat loss and fragmentation; however, some have suggested that interactions should be greater with loss. We updated the text to acknowledge this potential on Lines 77-79 and we updated Figure 1 to show predictions for both loss and fragmentation gradients.

Reviewer #1 (Remarks on code availability):

I did review the code and it looks ok. However, I expect major changes in the meta-analyses code, as I suggested in the comments for the authors.

>>>Response. Thank you for looking over the code. We have updated the code on the meta-analysis based on your comments.

Reviewer #2 (Remarks to the Author):

This manuscript presents a large-scale, long-term experiment manipulating habitat loss, fragmentation, and matrix quality in a factorial design, complemented by a meta-analysis of all existing experiments that concurrently manipulate habitat and matrix components. The authors use a focal host–plant specialist (Chelinidea vittiger) in a semi-natural system and integrate demographic modeling (POPAN) with patch-level abundance models to examine multi-scale population responses. The central claim is that landscape matrix quality can have effects on demographic rates and population size comparable to, or greater than, habitat loss and fragmentation, and that these effects are strongest when loss and fragmentation are low.
The topic is timely and relevant, and the authors address an important gap in the experimental literature. The experiment is ambitious in scope and represents one of the most comprehensive landscape manipulations available. The manuscript is clearly written and well framed in the context of ongoing debates in landscape ecology.

>>>Response. Thank you.

The authors have a strong track record working with this system, and this manuscript builds on several of their previous publications manipulating habitat configuration, studying dispersal, and examining matrix effects. While much of the conceptual framing and system-specific insights extend earlier work, this study offers clear empirical advances: (i) the simultaneous manipulation of habitat loss, fragmentation, and matrix quality in a fully factorial design; (ii) long-term demographic inference across 12 generations; and (iii) the first meta-analysis exclusively integrating experiments that manipulate both habitat and matrix characteristics. These represent incremental but meaningful contributions beyond the authors' prior research.

>>>Response. Thank you. In response to the Editor's comments, we added an additional statement in the Discussion on how this experiment ends prior work in this system.

Weaknesses
1. Generality is limited to a single specialist insect in a highly specific habitat-matrix system.

>>>Response. We fully appreciate this concern. In general, there is often a tradeoff between population-level and community-level inferences, whereby focusing on population-level data it can be possible to quantify mechanisms and detailed changes in populations, whereas at the community-level often the focus is on less detailed information (e.g., occupancy or species richness).

In this manuscript we focused on detailed demographic information, as most hypotheses for habitat loss, fragmentation, and matrix effects rely on demographic mechanisms (Fletcher et al. 2023a, Fletcher et al. 2024).

In Fletcher et al. (2023b) we show how occupancy responses to habitat loss and fragmentation were similar for other specialist insects in a parallel experiment in this system. Elsewhere, we have compared results of movement/connectivity with this species to some other vertebrates, finding qualitatively similar patterns (Fletcher et al. 2011, Fletcher et al. 2013). In general, we expect that this species/system is highly relevant to other specialist species that live in patchy environments, but clearly less relevant to species with broader habitat breadth or living in less patchy environments.

In this way, in the previous version of the manuscript we wrote: "…results are likely meaningful to other specialist species inhabiting fragmented landscapes (Fletcher et al. 2011, Fletcher et al. 2013). While our results provide key insights for wild populations, it remains to be seen if similar patterns arise at the community level, in terms of both species interactions and biodiversity metrics."

2. Some figures referenced (notably Fig. 2d–e) did not appear in the version reviewed.

>>>Response. Thank you for this note. 2d-e should have been in the original manuscript on the right side of Figure 2 (showing changes at the patch scale). In the revised manuscript, we increased the size of the labels and made sure they were included in the revision.

3. Interpretation of interactions among loss, fragmentation, patch size, and matrix quality is sometimes qualitative; effect sizes or partial dependence plots would improve clarity.

>>>Response. Thank you for this comment. For the experiment, in the Supporting Information, we provide all parameter estimates from the capture-recapture model and Figure 2 illustrates effect sizes. Partial responses are shown in Figure 2a, b, d, e.

For the meta-analysis, Figure 3 from the original manuscript showed pooled effect sizes based on the approach outlined in Gurevitch et al. (2000), what Macartney et al. (2022) call 'focal' effect sizes. Given this comment and other comments from reviewers, we extracted pair-wise partial effect sizes following Gurvetich et al. (2000) and Macartney et al. (2022). These pair-wise effect sizes are much easier to interpret, which we hope better addresses this concern.

4. Meta-analysis limitations: only five unique experiments are available, and several effect-size categories are dominated by single studies. This restricts statistical robustness and weakens quantitative generalizations.

>>>Response. We agree and were dismayed by how few experiments have been conducted that have manipulated both habitat and the matrix, but we do note that this information also helps illustrate the unique aspects of the experiment we show.

Figure 3 of the original manuscript showed sample sizes in terms of number of studies and number of effect sizes (which could be more than the number of species when a study reports several responses). This figure showed that for each test, there were at least 3 studies and for all tests reported except isolation, there were at least 20 effect sizes. However, we do recognize that these numbers are still limited and are pooling across response types (e.g., abundance, diversity, etc.). Despite this limited sample size, we think these effect sizes are still valuable as they provide a means to compare across the rigorous experiments that have been conducted to date. Furthermore, by reporting them, we hope that this will catalyze new experiments that fill key gaps we show.

5. Heterogeneity across studies in how "fragmentation" and "matrix quality" are defined complicates inference.

>>>Response. We agree. In particular, fragmentation was interpreted in two different ways: through manipulation of the number of patches and through manipulation of effective isolation. Matrix manipulations were more similar, in that matrix type was manipulated across the landscape and authors interpret these changes as changes in matrix quality (although the specific ways in which this was done varied across studies). We clarify these definitions on Lines 368-371 of the revised manuscript and provide some Discussion on Lines 186-189.

Statistical comments regarding the meta-analysis:
The meta-analysis is a useful addition but must be interpreted cautiously. The low number of studies, substantial heterogeneity in experimental manipulations, non-independence among multiple effect sizes per article, and the dominance of isolated experiments for some landscape elements all constrain the strength of the conclusions. It is surprising that confidence intervals shown in Fig. 3 for each moderator are identical while their sample sizes are different (especially for Fragmentation (Isolation) with a sample size of 9). While the multilevel meta-analytic framework is appropriate, the resulting estimates should be treated as qualitative patterns rather than firm quantitative benchmarks. The authors should temper claims about generality and acknowledge these statistical limitations more explicitly.

>>>Response. We checked our meta-regression modeling and are confident that these confidence intervals for this model were correct. However, the reason for this is that the model we used was aimed to extract effect size estimates for all tests (main effects and interactions) in a single model. Doing so means that the formulation implicitly assumed that heterogeneity across these main effects and interactions was the same.

To address this issue, we reformulated our meta-regression to allow for different amounts of heterogeneity across these treatment tests, which provides similar outcomes to testing each effect size separately. However, combining them in this way has some advantage to separate tests,

including that we can assess model diagnostics for one model rather than individual assessments of separate meta-regressions. We did so using a random slopes formulation. Resulting effect size estimates across tests now better capture the issue the reviewer raised.

In the revised Discussion, we also added some caution about the limitations of the meta-analysis on lines 191-196.

Reviewer #3 (Remarks to the Author):

This is a highly valuable study. In my opinion, it is unparalleled in its value for understanding the independent and interacting effects of habitat loss, fragmentation, and matrix quality on population abundance. Too often, these factors are either confounded or one (usually matrix quality) is simply ignored. Not only were these factors manipulated independently, but this was done at an appropriately large spatial scale. In addition, the authors were able to explain some of the underlying demographic mechanisms.

>>>Response. Thank you!

My main concern, which could probably be addressed easily, involves the topic of land sharing versus land sparing seemed. The authors did not clearly articulate what these terms, which may be unfamiliar to many ecologists, mean or how their study is relevant to these strategies for balancing food production with conservation. I found the concluding paragraph of the Discussion, which focuses on this topic, difficult to understand. The meaning of the first sentence in that paragraph is particularly unclear. To me, the topic does not seem directly relevant.

>>>Response. To keep clear, we simply removed the mention of sparing versus sharing in the conclusion paragraph. The first sentence of the conclusion paragraph was aimed at emphasizing that 'matrix' now dominates landscapes across the world. In the revision we simply remove this sentence as well.

Second, when interpreting the results of a statistical model and the interaction term for two variables is significant, it is fruitless to interpret what the results mean for the main effects of the variables involved in the interaction.

>>>Response. In terms of the interaction terms, we agree, although note that the extent to which it is an issue depends on whether interactions are orderly (change in magnitude) or disorderly (change in direction). We are not sure if the reviewer is referring to the reporting of our experimental results or the meta-analysis. For both, we report main effects for completeness, but focus on interactions.

In the revised manuscript, we also report pair-wise effect sizes in addition to the pooled effect sizes that were emphasized in the original manuscript (Fig. 3b). This reporting helps to better understand the form of interactions that were present in effect sizes.

References cited in response:

Astrom, J., and T. Part. 2013. Negative and matrix-dependent effects of dispersal corridors in an experimental metacommunity. Ecology **94**:72-82.

Fletcher, R. J., Jr., M. A. Acevedo, B. E. Reichert, K. E. Pias, and W. M. Kitchens. 2011. Social network models predict movement and connectivity in ecological landscapes. Proceedings of the National Academy of Sciences USA **108**:19282-19287.

Fletcher, R. J., Jr., M. G. Betts, E. I. Damschen, T. J. Hefley, J. Hightower, T. A. H. Smith, M. J. Fortin, and N. M. Haddad. 2023a. Addressing the problem of scale that emerges with habitat fragmentation. Global Ecology and Biogeography **32**:828-841.

Fletcher, R. J., Jr., A. Revell, B. E. Reichert, W. M. Kitchens, J. D. Dixon, and J. D. Austin. 2013. Network modularity reveals critical scales for connectivity in ecology and evolution. Nature Communications **4**:2572.

Fletcher, R. J., Jr., T. A. H. Smith, S. Troy, N. Kortessis, E. C. Turner, E. M. Bruna, and R. D. Holt. 2024. The prominent role of the matrix in ecology, evolution, and conservation. Annual Review of Ecology, Evolution, and Systematics **55**:423-447.

Fletcher, R. J., T. A. H. Smith, N. Kortessis, E. M. Bruna, and R. D. Holt. 2023b. Landscape experiments unlock relationships among habitat loss, fragmentation, and patch-size effects. Ecology **104**.

Peters, J. L., A. J. Sutton, D. R. Jones, K. R. Abrams, and L. Rushton. 2008. Contour-enhanced meta-analysis funnel plots help distinguish publication bias from other causes of asymmetry. Journal of Clinical Epidemiology **61**:991-996.

Viechtbauer, W., and M. W. L. Cheung. 2010. Outlier and influence diagnostics for meta-analysis. Research Synthesis Methods **1**:112-125.

Reviewer #1 (Remarks to the Author):

This paper will be a great contribution to the field. I still think, however, the meta-analysis is not changing (or reinforcing) the main results supported by the experiment. In fact, the current version is overselling the review/meta-analysis generality. For example, "We reviewed 2,221 articles..." This is actually the number of screened papers, and the results were extracted from 7 papers only.

>>>Response. Thank you. In the revised manuscript, most of the meta-analysis has now been moved to the Supplementary Information, which addresses the concerns raised.

Remarks on code availability:
The code works great and I tested it. No major problems. Easy to go through.

>>>Response. Thank you. We updated the code to be consistent with figure labeling in the revised text. It is now publicly available on Figshare.
